# An ant‐mimicking ant on an oceanic archipelago: *Camponotus guanchus* mimics *Crematogaster alluaudi*—An analogy with the situation of *Camponotus lateralis* (Hymenoptera: Formicidae)

**DOI:** 10.1002/ece3.70113

**Published:** 2024-08-19

**Authors:** Antonio J. Pérez‐Delgado, Herbert C. Wagner

**Affiliations:** ^1^ Island Ecology and Evolution Research Group Institute of Natural Products and Agrobiology (IPNA‐CSIC) La Laguna Spain; ^2^ School of Doctoral and Postgraduate Studies University of La Laguna La Laguna Spain; ^3^ Institute of Biology University of Graz Graz Austria

**Keywords:** Batesian mimicry, Canary Islands, *Gallotia*, *Perenotus*, regional color morphs

## Abstract

Mimicry, that is, the imitation of any unpalatable or defensive species by another, has been of central interest to evolutionary research since Darwin's lifetime. Two ant species, *Camponotus guanchus* Santschi, 1908 and *Crematogaster alluaudi* Emery, 1893, endemic to the Canary Islands, occur in two color‐morphs: While the head of workers is always reddish and the gaster blackish, the mesosoma (inclusive waist) is either fully reddish or fully blackish. In addition to the obvious morphological and coloration similarities, we provide evidence of mimicry: (i) *Ca. guanchus* was found only within the area of *Cr. alluaudi*. (ii) Color morphs are geographically non‐randomly distributed: Workers of both species from 16 localities of syntopic occurrences shared in eight cases a blackish and in eight cases a reddish mesosoma. Hence, *Ca. guanchus* mimics both local color‐morphs of *Cr. alluaudi*. We consider a fascinating analogy with the Mediterranean mimicry system in *Camponotus lateralis* (Olivier, 1792) and its model species of the *Crematogaster scutellaris* (Olivier, 1792) group on an island scale. Additionally, we present two endemic bug species, *Perenotus stysi* (Ribes et al., 2008) and *P. malobae* Roca‐Cusachs & Goula, 2016, as mimics of those *Cr. alluaudi* workers having a reddish mesosoma. Our distribution, coloration, frequency, and behavioral data as well as the analogy with *Ca. lateralis* and the *Cr. scutellaris* group suggest a Batesian‐mimicry system in which *Ca. guanchus*, *Perenotus stysi*, and *P. malobae* mimic the unpalatable and aggressive *Cr. alluaudi* as an antipredator adaptation.

## Graphical abstract

Two Canarian ant species, *Camponotus guanchus* Santschi, 1908 (a, c) and *Crematogaster alluaudi* Emery, 1893 (b, d), occur in two color‐morphs, one with a reddish (a, b) and the other with a blackish (c, d) mesosoma. Color morphs are geographically non‐randomly distributed but *Ca. guanchus* mimics both local color‐morphs of the unpalatable and aggressive *Cr. alluaudi*.

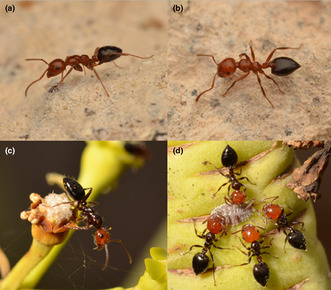

## 1 INTRODUCTION

Shortly after Charles Darwin postulated the theory of natural selection (Darwin, 1859), Henry W. Bates described Batesian mimicry in Lepidoptera. He found that species with obvious similarities in color were not always closely related. Most Lepidopteran species with striking colors were toxic and thus unpalatable for insectivores (aposematism sensu Poulton, 1890). Some palatable species mimicked the color of toxic species as an antipredator adaptation (Bates, 1861). From that time on, mimicry has remained a topic of central interest for evolutionary research (Darwin, 1871; Fisher, 1930; Komárek, 2002; Quicke, 2017; Ruxton et al., 2004; The Heliconius Genome Consortium, 2012; Wallace, 1866; Wickler, 1968). In Batesian mimicry, the morphology and color of mimics resembles that of any unpalatable or defensive species (i.e., the model); mimics enjoy reduced predation without the need to evolve a costly defense like a toxin or sting (Franks & Noble, 2004; Wickler, 1968). In Muellerian mimicry, several nonclosely‐related unpalatable or defensive species share an honest aposematic signal to warn predators (Mallet & Gilbert, 1995; Müller, 1879). In so doing, fewer individuals from each species are attacked by experienced predators and, consequently, mutually benefit (Mallet & Gilbert, 1995; Symula et al., 2001; Wilson et al., 2015).

Since ants are often well‐fortified against, aggressive towards, or unpalatable for potential predators (Hölldobler & Wilson, 1990), they represent models for thousands of Batesian mimetic species from different arthropod orders, including Hemiptera, Coleoptera, non‐ant Hymenoptera, Diptera, and Araneae (e.g., McIver, 1987; Oliveira & Sazima, 1984; Pekár, 2020; Pekár & Křál, 2002; Pie & Del‐Claro, 2002; Rasekh et al., 2010; Reiskind, 1977; Taniguchi et al., 2005). Mimicry among ants is rare and a hitherto little‐studied topic, only less than 20 examples of ant species mimicking other ant species are known worldwide (Emery, 1886; Fisher & Peeters, 2019; Forel, 1874, 1891; Gallego Ropero & Feitosa, 2014; Gobin et al., 1998; Ito et al., 2004; Menzel et al., 2010; Merrill & Elgar, 2000; Pekár et al., 2017; Powell et al., 2014; Rasoamanana et al., 2017; Schifani et al., 2022; Seifert, 2019; Wagner, 2013, 2014, 2019; Ward, 1984, 2009). Since mimicry can only be adaptive in the presence of a model, mimic and model should occur together in time and space. Hence, it is accepted that the distribution area of mimics is usually within those of its model (Kunte et al., 2021; Merrill & Elgar, 2000; Pekár et al., 2017; Wilson et al., 2015).

The Canary Islands are an archipelago of volcanic origin, formed by seven main islands about 90 km off the northwest coast of the African continent. With an area of 7492 km^2^, the Canary Islands have 7820 invertebrate species (45% endemics), of which 61 are ant species (31% endemics) (Gobierno de Canarias, 2024). In general, the origin and distance from the continent have turned oceanic islands into “laboratories” for the study and understanding of evolutionary processes (Gillespie & Roderick, 2002; Graham et al., 2017; Whittaker & Fernandez‐Palacios, 2007). The biota of these islands are characterized by: (i) having fewer species per area unit than those on the continent, (ii) having a different balance of species compared to areas with an equivalent size on the continent (island disharmony), and (iii) a high number of endemic species (Whittaker & Fernandez‐Palacios, 2007).

**FIGURE 1:**
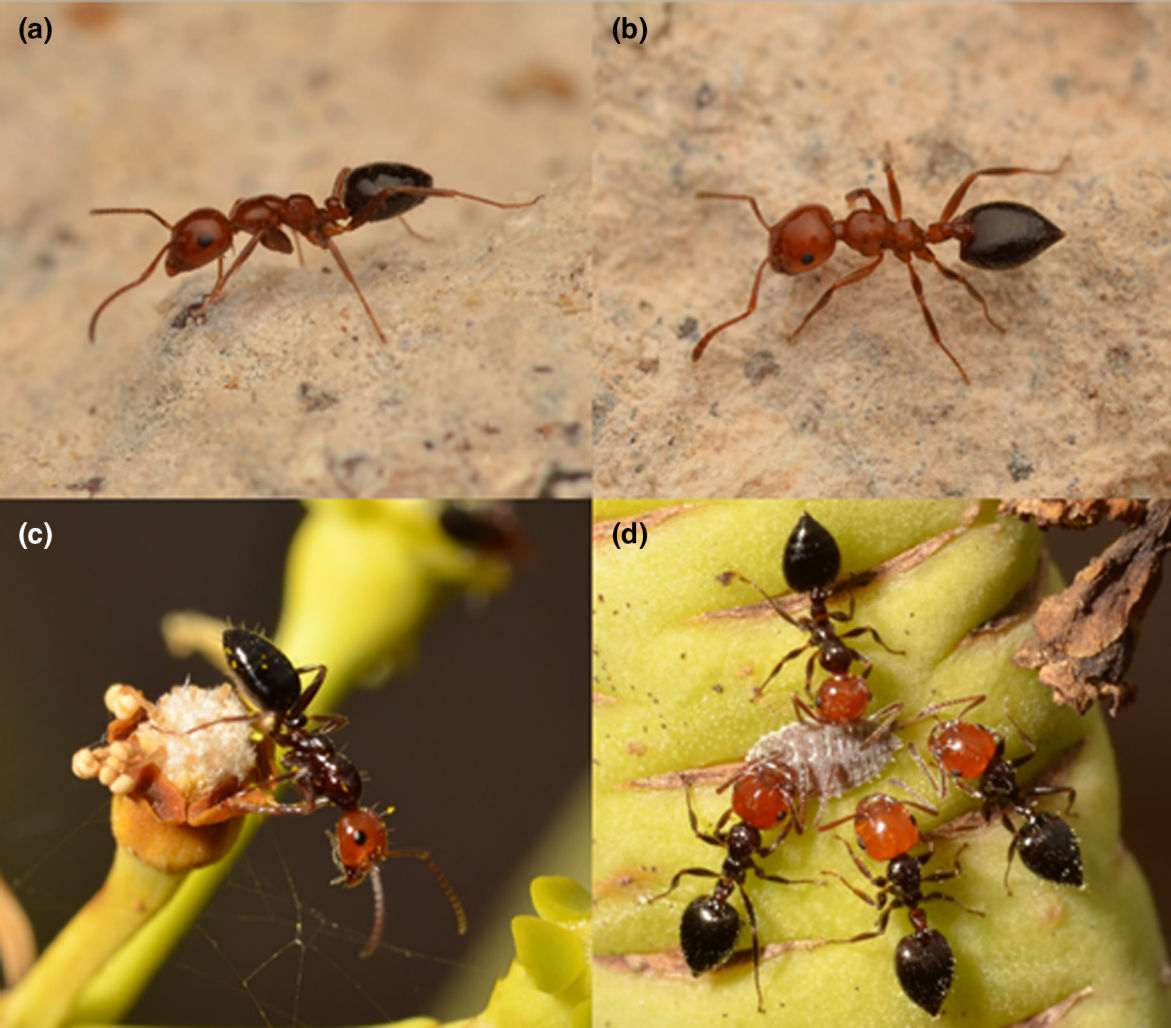
Workers of *Camponotus guanchus* and *Crematogaster alluaudi* with different mesosoma colors. (a) *Ca. guanchus* and (b) *Cr. alluaudi* workers with a reddish mesosoma collected on La Palma, Monte de Luna, 02/03/2021; (c) *Ca. guanchus* and (d) *Cr. alluaudi noualhieri* workers with a blackish mesosoma collected on Tenerife, Punta del Hidalgo, 17/07/2021. Individuals deposited in the A. J. Pérez‐Delgado collection.

**FIGURE A1:**
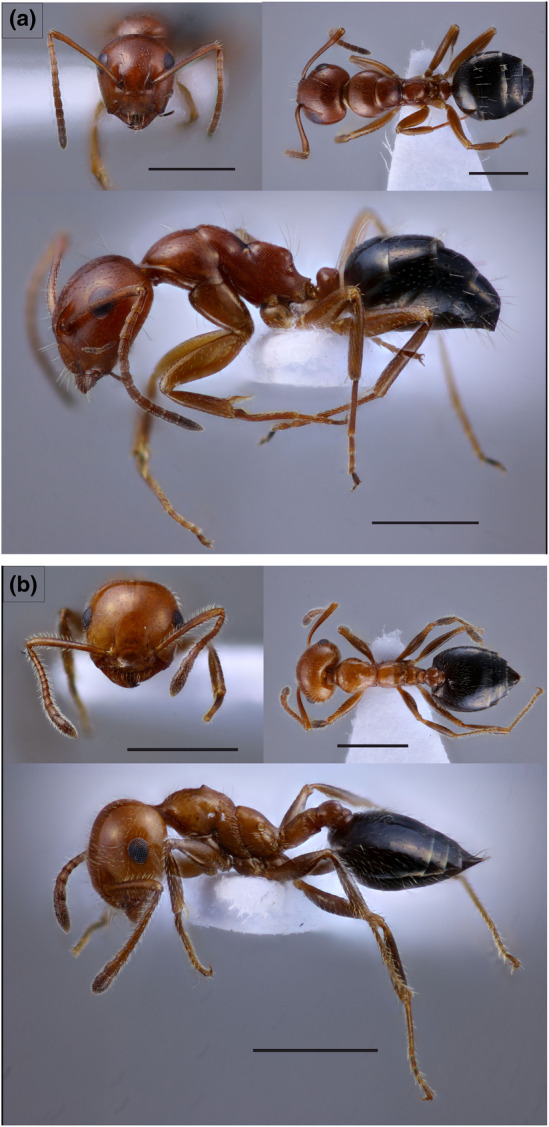
*Camponotus guanchus* worker (a) and *Crematogaster alluaudi* worker (b). Detail of the head, dorsal view, and lateral view of both species. The scale is equivalent to 1 mm.

**FIGURE A2:**
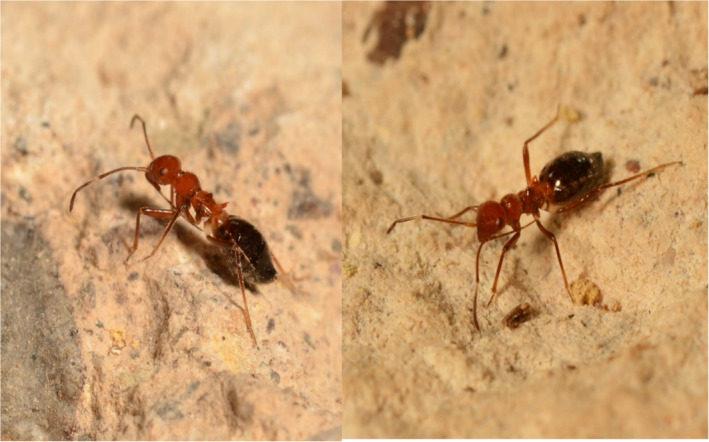
Habitus of *Perenotus malobae*—a myrmecomorphic Heteroptera species mimicking *Crematogaster alluaudi* in color and morphology. Specimen collected at Monte de Luna, La Palma. Individual deposited in the Pérez‐Delgado collection.

The mimicry system we propose is formed by two ant species, *Camponotus guanchus* Santschi, 1908 and *Crematogaster alluaudi* Emery, 1893, as well as by the mirid bug genus *Perenotus*. They are endemic to the Canary Islands (Barquín Diez, 1981; Cagniant & Espadaler, 1993; Emery, 1925; Roca‐Cusachs et al., 2020; Wellenius, 1955) and their mimicry is based on morphology and color (Figure 1; Figures A1 and A2 in Appendix).

*Camponotus guanchus* (Formicinae) was known to occur on the islands of El Hierro, La Palma, and Tenerife to date (Gobierno de Canarias, 2024), in this publication we provide data also for the island La Gomera (Table A1 in Appendix). This species has two color variants, which we call “color morphs.” While the head of both morphs is reddish and the gaster blackish, the mesosoma (including the waist) is either entirely reddish (reddish morph) or entirely blackish (blackish morph) (Figure 1). Following the current taxonomic status, both color morphs belong to the same species (Cagniant & Espadaler, 1993). *Camponotus guanchus* was described from the north of Tenerife (Santschi, 1908) and the holotype has a blackish mesosoma (see AntWeb, CASENT0911695). We are not the first to have noticed the similarity between *Ca. guanchus* and *Cr. alluaudi*: Santschi (1919) mentioned color mimicry in *Ca. guanchus* and Emery (1893) compared the relationship between *Ca. guanchus* and *Cr. alluaudi* with that between *Ca. lateralis* (Olivier, 1792) and *Cr. scutellaris* (Olivier, 1791) in the Mediterranean. However, these authors only reported about the blackish morphs. *Camponotus guanchus* might be very closely related to *Ca. ruber* Emery, 1925, a taxon occurring in North Africa and South Europe (Cagniant, 1996; Schifani et al., 2022; Seifert, 2019). Although the unique petiole shape in *Ca. guanchus* workers makes a putative synonymy with this taxon unlikely (Cagniant & Espadaler, 1993), the possibility of a synonymy should be proven by a modern taxonomic‐revision including morphometric or nuclear molecular‐genetic data.

*Crematogaster alluaudi* (Myrmicinae) is suggested to be the model species of this mimicry system. It is territorial and very aggressive (personal observations). Workers, if alerted, fold their gaster up above the mesosoma in a scorpion‐like manner (Barquín Diez, 1981). All *Crematogaster* species secrete a defensive venom, produced by the Dufour's gland, when disturbed (Buren, 1959; Daloze et al., 1987, 1991). This venom can kill other ants rapidly (Marlier et al., 2004; Seifert, 2018) and possibly makes them unpalatable to vertebrate predators (Emery, 1886; Ito et al., 2004; Wagner, 2013, 2014). *Crematogaster alluaudi* is distributed over four of seven islands of the archipelago: El Hierro, La Palma, La Gomera, and Tenerife (Gobierno de Canarias, 2024; Table A1 in Appendix). This species appears in the same two color‐morphs as *Ca. guanchus* (Figure 1). Its subspecies *Cr. alluaudi noualhieri* Emery, 1893 is restricted to the northern slope of the island Tenerife. Its blackish mesosoma‐color is the only distinct difference from the nominate form (Santschi, 1937). The evaluation of putative small morphological differences (Barquín Diez, 1981; Emery, 1893) would require morphometric data. Since the status as subspecies is taxonomically doubtful, we use here the term “color morph”.

Another putative member in the mimicry system, that we briefly introduce here, is the endemic bug genus *Perenotus* (Hemiptera: Miridae) with two known species, *P. stysi* (Ribes et al., 2008) from Tenerife and *P. malobae* Roca‐Cusachs & Goula, 2016 from La Palma. These two species were already mentioned to be myrmecomorphic, but no model ant‐genus has been suggested yet (Ribes et al., 2008; Roca‐Cusachs & Goula, 2016). Both species of the genus *Perenotus* have a reddish head, a reddish thorax, and a blackish abdomen (Figure A2 in Appendix; Ribes et al., 2008, Roca‐Cusachs & Goula, 2016).

Analogous to the situation in *Ca. guanchus* and *Cr. alluaudi*, also the Mediterranean ant *Ca. lateralis* (Olivier, 1792) and its *Crematogaster* model species have regional mesosoma‐color differences. In some regions, for example, over large parts of the Balkans, *Ca. lateralis* has a reddish mesosoma like *Crematogaster schmidti* (Mayr, 1853). In other regions, for example, over large parts of Italy, it has a blackish one like *Cr. scutellaris* (Olivier, 1791). It has already been hypothesized that the submissive (Borovsky et al., 2022; Marlier et al., 2004; Menzel et al., 2010) *Ca. lateralis* mimics the color of the regional occurring dominant and very aggressive *Crematogaster* model species (Seifert, 2018, 2019; Wagner, 2013, 2014).

This study aims to demonstrate evidence of mimicry of *Ca. guanchus* beyond the obvious resemblance to *Cr. alluaudi*. For this purpose, we analyzed the geographic distribution of *Ca. guanchus* and *Cr. alluaudi* and their putative mesosoma‐color coincidence in sympatric occurrences. We hypothesize that the two color‐morphs of *Ca. guanchus* mimic the local color‐morphs of *Cr. alluaudi*. The alternative hypothesis is that there is no mimicry between these species. This option would be valid if the distribution of *Ca. guanchus* (and its color morphs) was not linked to the distribution of *Cr. alluaudi* (and its color morphs) but distribution as well as the matching of color‐morphs was random. We expect *Perenotus* to occur only in regions where *Cr. alluaudi* workers share its reddish mesosoma.

## 2 MATERIALS AND METHODS

Distribution data of *Ca. guanchus*, *Cr. alluaudi*, and *Perenotus* spp. were obtained from the collection of Antonio J. Pérez‐Delgado and the citizen‐science platform iNaturalist (https://www.inaturalist.org/), the material was collected between 2001 and 2023 (Table A2 in Appendix). All available *Ca. guanchus*, *Cr. alluaudi*, and *Perenotus* spp. records were considered for distribution maps (QGIS Development Team, 2019) but the statistical analysis was performed with the information from the database only (IBM SPSS Statistics 19). *Perenotus* records were not tested statistically due to their low number. The behavior of ants was observed directly in the field.

To test if *Ca. guanchus* follows the island distribution of *Cr. alluaudi* rather than those of other Canarian ant‐taxa (which were predicted to be outside of the mimicry system), island distribution of *Ca. guanchus*, *Cr. alluaudi*, and all other Canarian ant‐taxa were listed (Table A1 in Appendix) based on literature information (Gobierno de Canarias, 2024) and own collecting activity. The percentage of native ant‐species outside the mimicry system sharing the island distribution of *Cr. alluaudi* is considered as *p*‐value to estimate how probable the shared island‐distribution of *Ca. guanchus* and *Cr. alluaudi* is a random result.

To evaluate the putative geographic coincidence of mesosoma colors between the two species, a matrix including the cases in which both species were in syntopy was established (Table 1). Fisher's exact test (Fisher, 1922, 1925), a conservative statistical method (Crans & Shuster, 2008), was used to test how probable the common occurrences of the same color‐morphs of both species were a random result. The one‐tailed *p*‐value was accepted. An α‐level of .05 was used.

**TABLE 1:** Syntopic occurrences of color‐morphs of *Ca. guanchus* and *Cr. alluaudi* on the Canary Islands.

	*Ca. guanchus* with reddish mesosoma	*Ca. guanchus* with blackish mesosoma
*Cr. alluaudi* with reddish mesosoma	8	0
*Cr. alluaudi* with blackish mesosoma	0	8

Images of the habitus were taken using a Canon EOS 750D attached to a Zeiss Stemi 2000 stereomicroscope, multiple images were processed with Zerene Stacker (V.1.04, Zerene Systems, LLC., Richland, USA) to generate an improved single image using pmax and dmap methods. Additionally, more specimens were photographed in their habitat using a NIKON D5100 and objective Sigma 105 mm.

## 3 RESULTS

**FIGURE A3:**
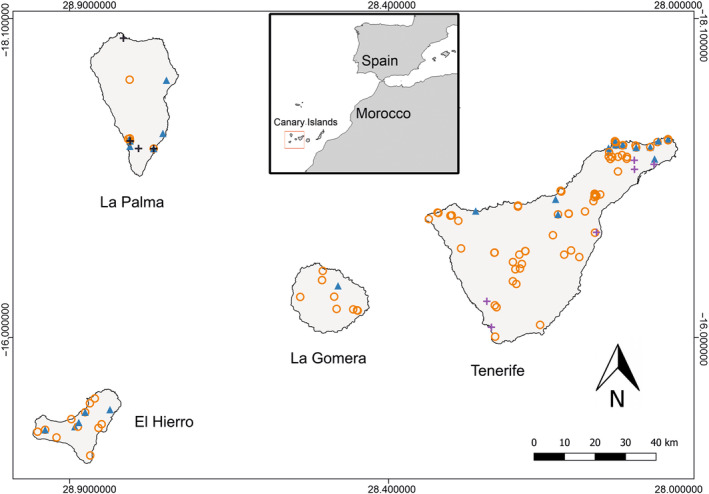
Distribution of the mimicry‐system members on Canary Islands: *Crematogaster alluaudi* (circles), *Camponotus guanchus* (triangles), *Perenotus malobae* (black crosses), and *Perenotus stysi* (purple crosses).

From 930 localities throughout the Canary archipelago included in the collection of Pérez‐Delgado, *Ca. guanchus* and *Cr. alluaudi* were found at 90; we added also 39 records from iNaturalist (https://www.inaturalist.org/) to get a total of 129 records from the islands El Hierro, La Palma, La Gomera, and Tenerife. Ten records of *Perenotus* on the islands Tenerife and La Palma were known (see Figure A3 in Appendix).

Of 59 Canarian ant‐taxa outside the mimicry system, 33 were native to the Canary Islands (see Table A1 in Appendix) (Gobierno de Canarias, 2024). Based on our presence list of ants of the seven Canary Islands (Table A1 in Appendix), only one taxon (3.0%) resembles the island distribution of *Cr. alluaudi* and *Ca. guanchus*: *Temnothorax gracilicornis* (Emery, 1882) is the only native species outside the mimicry system which occurred also only on the four western but not on the three eastern islands.

*Camponotus guanchus* was found at an altitude of 279 ± 200 (min = 35, max = 686) m a.s.l. (Table A2 in Appendix), the most frequent habitat was the thermo‐sclerophyllous woodland (60%) (Table A2 in Appendix). *Crematogaster alluaudi* has been found in a wider vertical range from coastal to high mountain habitats (Table A2 in Appendix) at 716 ± 664 (min = 19, max = 2378) m a.s.l., the most frequent habitat was also thermo‐sclerophyllous woodland (34%) (Table A2 in Appendix).

**FIGURE 2:**
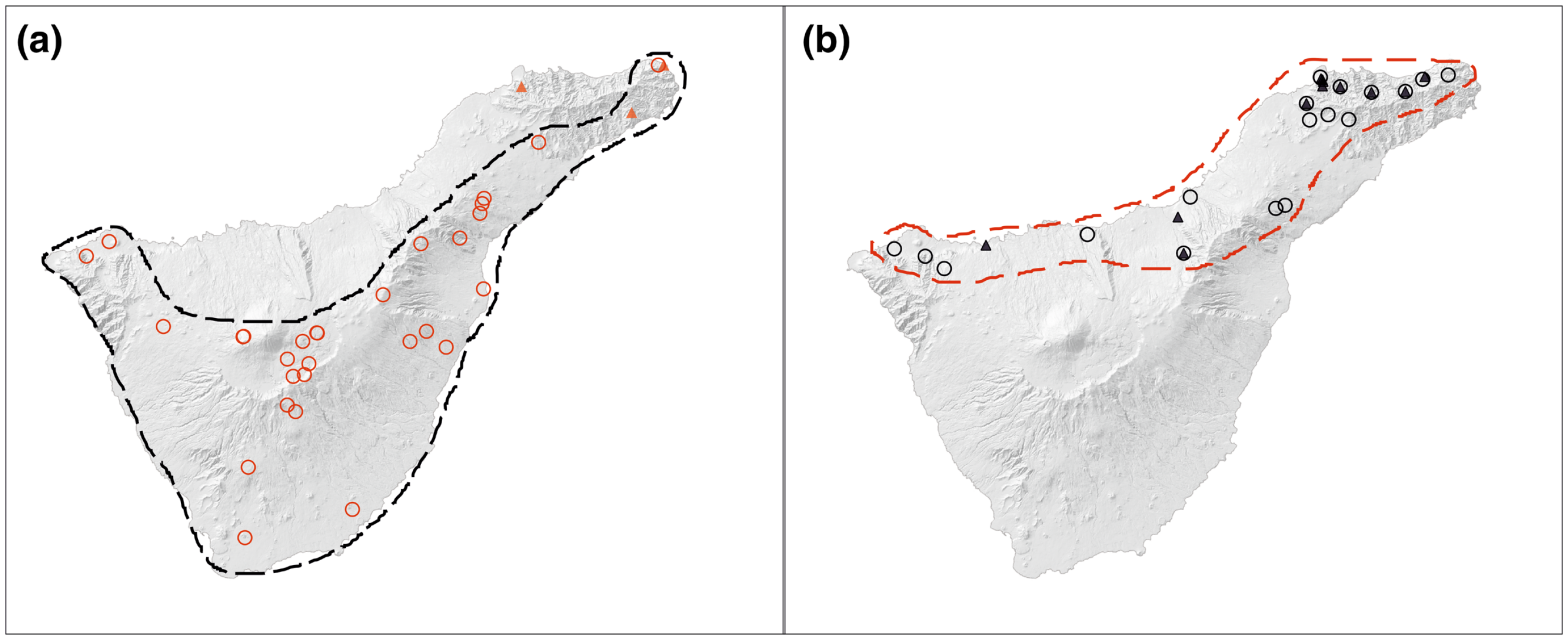
Distribution of color morphs of *Camponotus guanchus* and *Crematogaster alluaudi* on the island Tenerife. The circles correspond to *Cr. alluaudi* and the triangles *Ca. guanchus*. (a) Reddish color‐morphs and (b) blackish color‐morphs of both species.

*Camponotus guanchus* was recorded at 30 localities (70 workers in total), at 16 (53%) with a reddish and at 14 (47%) with a blackish mesosoma (Table A2 in Appendix). *Crematogaster alluaudi* occurred at 99 localities (524 workers in total), at 62 (63%) with a reddish and at 37 (37%) with a blackish mesosoma (Table A2 in Appendix). All observed workers had either an entirely reddish or blackish mesosoma (Figure 1), no worker with intermediate coloration was found. No intracolonial coexistence of the two color‐morphs was detected either. The reddish color‐morphs were found on all four islands, those with a blackish mesosoma only on the northern slope of Tenerife (Figure 2; Figure A3 and Table A2 in Appendix). Tenerife is the only island from where both color‐morphs of both species were recorded. The distribution areas of color morphs of *Ca. guanchus* were within those of the equivalent color morph of *Cr. alluaudi* (Figure 2). A single worker of the reddish color‐morph of *Ca. guanchus* (cod. 3052_TF‐345) broke this rule (Figure 2a). In our study, at 99 sample points *Cr. alluaudi* (any color morph) was found and at 16 of them also *Ca. guanchus* was detected. In eight of these cases, both species had a blackish and in further eight cases a reddish mesosoma (for details see Table A2 in Appendix). No locality with a mesosoma‐color difference between *Ca. guanchus* and *Cr. alluaudi* was detected (Table 1). The probability for the described color‐pattern between the two species to be a random result is *p* = .0001. When both species occurred syntopically, *Ca. guanchus* was always in the minority and did not exceed 10% of individuals of *Cr. alluaudi* workers. The workers of both species were commonly observed foraging on the same plant. We observed three times weakly‐used mixed trails of both species leading to flowers of *Euphorbia lamarckii*; workers of *Ca. guanchus* escaped when they encountered a *Crematogaster* worker. Two times *Cr. alluaudi* workers were present at the flowers and *Ca. guanchus* workers kept distance.

Including literature data, there are 10 localities where the genus *Perenotus* was detected so far (Ribes et al., 2008; Roca‐Cusachs & Goula, 2016). We found one individual within a nest of *Cr. alluaudi*. All individuals shared the distribution as well as the color pattern (Figures A2 and A3 in Appendix) with *Cr. alluaudi* workers having a reddish mesosoma (Figure A2 in Appendix). From localities in the north of Tenerife, where workers of *Cr. alluaudi* had a blackish mesosoma, no *Perenotus* records were made.

## 4 DISCUSSION

We demonstrated the hypothesis of mimicry between *Ca. guanchus* and *Cr. alluaudi* based on the following findings:

### 4.1 Sympatric occurrence

The species *Ca. guanchus* and *Cr. alluaudi* were both found only on the four western islands (El Hierro, La Palma, La Gomera, and Tenerife). The fact that this horizontal distribution‐pattern in the archipelago is shared with only one single ant‐species outside the mimicry system (*Temnothorax gracilicornis*) indicates a rare occurrence. Among our target species, *Cr. alluaudi* has a wide vertical distribution ranging from coastal to high mountain habitats, the most frequent habitat is thermo‐sclerophyllous woodland. *Camponotus guanchus* has a narrower niche, often limited to thermo‐sclerophyllous woodland, *Euphorbia* scrub, and shrublands (del Arco Aguilar & Delgado, 2018; 93% of observations). Therefore, we consider the horizontal and vertical distribution of *Ca. guanchus* to be fully within that of *Cr. alluaudi* (Figure 2; Figure A3 and Table A2 in Appendix).

### 4.2 Same color morphs occur syntopically

While the morphs with the reddish mesosoma of both species occurred on all four islands, we found workers with a blackish mesosoma in both species only on Tenerife. Our results show that the color morphs of both species were geographically segregated: The blackish morph of both species occurred on the northern slope of the island and the reddish morph on the rest of the island (Figure 2). The results allowed us to detect contact zones in which the different color‐morphs were close together (Figure 2; Table A2 in Appendix). We have detected only one outlier contradicting the spatial structure of the color morphs: One specimen of *Ca. guanchus* with a reddish mesosoma was detected within the area of the blackish color‐morph (Figure 2a; Table A2 in Appendix). Since this specimen was found in an anthropized area of vegetal‐waste deposit, there is a possibility that it was introduced.

The results show that, when both species were in syntopy, their mesosoma colorations were not random. We found a significant local congruence between the mesosoma colors of *Ca. guanchus* and *Cr. alluaudi* when we analyzed the results at the archipelago level. If we focused on the island Tenerife, we have found the species in syntopy nine times and in 100% of cases the color morph of the mimic coincided with that of the model. Due to the low number of samples on island level, we have not been able to do statistical analyses but still the mimicry seems obvious. Since we have not detected intermediate‐colored workers or colonies with both color‐morphs in *Cr. alluaudi* as well as *Ca. guanchus*, we believe that inter‐color mating is not common. Color coincidence is a key fact that confirms our hypothesis of mimicry. This result makes us believe that *Ca. guanchus*, fascinatingly, represents a mimic with a similar evolutionary history as the not very closely related (Seifert, 2019) species *Ca. lateralis*: Local color‐morphs are hypothesized to occur in dependence on the color of *Crematogaster* models (Seifert, 2018, 2019; Wagner, 2013, 2014). While the mimicry system of *Ca. lateralis* extends over an area of more than 1,000,000 km^2^ (Mediterranean, Black Sea Region, Anatolia, and Caucasus), the mimicry system of *Ca. guanchus* extends over an insular area of only ca. 3300 km^2^. In the *Ca. lateralis* mimicry‐system, there are three very closely related (Blaimer, 2012) model species (*Cr. scutellaris*, *Cr. schmidti*, *Cr. ionia* Forel, 1911), which also hybridize at least partly (Bračko, 2023; Seifert, 2018), but only one mimic species (*Ca. lateralis*) (Seifert, 2019). In contrast, the mimicry system on the Canary Islands includes probably a single model‐species, *Cr. alluaudi*, with two color morphs. Moreover, *Ca. ruber*, a further Mediterranean taxon very similar in color and morphology to *Ca. guanchus* and putatively closely related (if not conspecific), has also been suggested to be a mimic of *Cr. scutellaris* (Schifani et al., 2022). Could *Ca. guanchus* be derived from a species mimicking the *Cr. scutellaris* group? In the case of *Cr. alluaudi*, the absence of propodeal spines (Figure A1b in Appendix; Emery, 1893) and nest sites occurring in dead succulent chamaephytes (own observations: *Kleinia neriifolia*, *Aeonium arboreum*) but also in soil under stones (Barquín Diez, 1981; Wellenius, 1955) make a close relatedness to the three arboricolous Mediterranean species of the *Cr. scutellaris* group unlikely. Anyway, molecular‐genetic studies should investigate the putative evolutionary relatedness of *Camponotus* and *Crematogaster* species belonging to the described mimicry systems. A further similar Mediterranean ant‐mimicry example are the two species of *Colobopsis*, *Co*. *truncata* (Spinola, 1808) and *Co. imitans* Schifani et al., 2022, which mimic *Dolichoderus quadripunctatus* Linnaeus, 1771 and *Cr. scutellaris*, respectively, in terms of color. Both mimics are closely related to each other and morphologically cryptic, color is the most obvious difference between them. It has been suggested that the presence of different models in different parts of Europe has driven their color mimicry and thus their speciation process (Schifani et al., 2022). It seems possible that intraspecific color‐morphs of *Ca. guanchus* or *Ca. lateralis* are at an earlier evolutionary stage of speciation than the two *Colobopsis* species.

Trail‐sharing behavior with *Crematogaster*, which was often observed in *Ca. lateralis* (Baroni Urbani, 1969; Carpintero et al., 2005; Goetsch, 1942, 1951; Kaudewitz, 1955; Menzel et al., 2010, 2014), occurs also in *Ca. guanchus*. *Camponotus lateralis* workers escape when they encounter a *Crematogaster* worker (Kaudewitz, 1955). Analogously, we observed workers of *Ca. guanchus* in shared trails escaping when they encountered a *Cr. alluaudi* worker. Due to the low abundance of *Ca. guanchus*, unfortunately, we made only few observations and definite observation sets lack.

The genus *Perenotus* is a further member of the proposed mimicry system. Its morphology, coloration, and distribution coincide with that of the reddish morph of *Cr. alluaudi*. Furthermore, the presence of one individual of *P. malobae* inside a nest of *Cr. alluaudi* underpins the mimicry hypothesis. All these findings raise questions concerning the putative unpalatability of *Cr. alluaudi* for predators.

### 4.3 Predation pressure as a driver for color change

Drivers of the evolution of color change should be searched among animals perceiving reddish color, such as lizards (Benes, 1969; Wagner, 1932), birds (Hess, 1917; Lashley, 1916; Yerkes & Eisenberg, 1915), or jumping spiders (Zurek et al., 2015). The diversity of lizards and birds in the distribution area of *Cr. alluaudi* and *Ca. guanchus* is low (Gobierno de Canarias, 2024). There is little information about the diet of birds, but a few studies (Padilla et al., 2005; Rodríguez et al., 2007) and unpublished observations by local ornithologists (see Acknowledgments) could suggest that the frequency of predation on ants is low. However, there is more information on the diet of lizards. The only lizards sharing island distribution with our target ant‐species belong to the Canarian‐endemic genus *Gallotia*: *Gallotia caesaris caesaris* (Lehrs, 1914) on El Hierro, *Gallotia caesaris gomerae* (Boettger & Müller, 1914) on La Gomera, *Gallotia galloti palmae* (Boettger & Müller, 1914) on La Palma, *Gallotia galloti eisentrauti* Bischoff, 1982 in the north of Tenerife, and *Gallotia galloti galloti* (Oudart, 1839) on the rest of Tenerife (Gobierno de Canarias, 2024). They are omnivorous (Novosolov & Meiri, 2013; Rodríguez et al., 2008; Valido & Nogales, 2003). Remains of ants were found in 25%–46% of the *Gallotia galloti* droppings investigated and ants represented a percentage that varied between 30% and 74% of the total insect individuals consumed (Rodríguez et al., 2008; Valido et al., 2003). One could suspect whether such a tiny number of potential vertebrate predator taxa is sufficient to drive the evolution of aposematism and mimicry. However, a high average density of individuals (density compensation) compensates this low vertebrate species richness of insular ecosystems (Whittaker & Fernandez‐Palacios, 2007). Density compensation refers to a higher‐than‐normal density related to empty‐niche phenomena and reduced predation (Novosolov & Meiri, 2013; Whittaker & Fernandez‐Palacios, 2007). Several studies have estimated maximal densities of *Gallotia galloti* on Tenerife at 1300–3300 individuals/ha (De Los Santos & De Nicolás, 2008; Farina & Aguilar, 2002). To summarize a conclusion, we interpret predation pressure by *Gallotia* lizards to be high enough to drive of evolution in the proposed mimicry‐system. This interpretation, however, presumes that *Gallotia* should avoid the model of the mimicry system, most likely *Cr. alluaudi*.

### 4.4 Is the mimicry of *Ca. guanchus* Batesian?

It is believed that predation pressure drives the evolution of aposematism (Poulton, 1890; Quicke, 2017; Wickler, 1968). By means of aposematism, *Cr. alluaudi* might defend itself against sympatric predators which perceive reddish color. The color pattern would be particularly suitable for an optical differentiation from other ants. Nowadays, the classical categorization (Wickler, 1968) into Batesian (Bates, 1861) versus Muellerian (Müller, 1879) mimicry is considered to represent a simplification (Speed, 1999). Instead, there is a mimetic spectrum of species that vary in their honesty and their degree of unpalatability or defensiveness between the lower and upper extreme, that is, Batesian and Muellerian mimicry (Pekár et al., 2017). We argue that *Ca. guanchus* might be close to the lower extreme of this spectrum, that is, in the classical sense (Wickler, 1968), Batesian mimicry. The alternative hypothesis would be that predators do not have a preference for *Ca. guanchus* or *Cr. alluaudi*, and instead are repelled by both (e.g., the formic acid of *Ca. guanchus* and the venom of *Cr. alluaudi*). If this alternative hypothesis was true, the color signals would represent Muellerian mimicry (Müller, 1879; Wilson et al., 2015). In the following, we present arguments for an allocation of *Ca. guanchus* within the classic concept of Batesian and Müllerian mimicry:

1. In syntopic occurrence, the frequency of *Ca. guanchus* workers did not exceed 10% of that of *Cr. alluaudi*. Being in the minority compared to individuals of the model is typical for Batesian but not for Muellerian mimics (Kikuchi & Pfennig, 2010; Wickler, 1968).
2. The distribution area of *Ca. guanchus* is embedded within those of *Cr. alluaudi* while the latter has a wider ecological niche (Figure 2; Figure A3 and Table A2 in Appendix). This finding is in line with most other cases of Batesian ant‐mimicking ants, where models had a larger area than their mimics (Gallego Ropero & Feitosa, 2014; Ito et al., 2004; Merrill & Elgar, 2000; Schifani et al., 2022); theoretically, Batesian mimics are expected to co‐occur with their models (Kunte et al., 2021; Pfennig & Mullen, 2010).
3. Workers of *Ca. guanchus* follow trails of *Cr. alluaudi* and, consequently, seek their physical closeness (which should be adaptive for a Batesian mimic). Vice versa, *Cr. alluaudi* behaves aggressively to *Ca. guanchus*, which should be adaptive for a model to avoid physical closeness to a mimic weakening the honesty of the aposematic signal (Kikuchi & Pfennig, 2013).
4. In the analogous mimicry system in the Mediterranean, represented by *Ca. lateralis* and the *Cr. scutellaris* group, it was shown that Italian wall lizards (*Podarcis sicula*) ingest highly significant more *Ca. lateralis* group workers than those of the *Cr. scutellaris* group (Wagner, 2013, 2014; H. C. Wagner et al., in prep.). We expect that the defense chemicals of ants in the here described Canarian mimicry‐system are similar. More generally, even if ant acid of *Camponotus* might be unpalatable for some predators, formicine ants may be relatively more vulnerable to predation than those of other subfamilies (Lamon & Topoff, 1981; Merrill & Elgar, 2000; Montgomery, 1985; Seifert, 2009).

To summarize, we suggest that the color of the mimic *Ca. guanchus* resembles that of the unpalatable species *Cr. alluaudi* as an anti‐predator adaptation, that is, Batesian mimicry (Bates, 1861). In other words: Within a mimetic spectrum of defense (Pekár et al., 2017), *Ca. guanchus* is close to the lower extreme of unpalatability or defensiveness.

## AUTHOR CONTRIBUTIONS

**Herbert C. Wagner:** Conceptualization (equal); formal analysis (equal); funding acquisition (lead); investigation (equal); methodology (equal); project administration (equal); resources (equal); validation (equal); visualization (equal); writing – original draft (equal); writing – review and editing (equal). **Antonio J. Pérez‐Delgado:** Conceptualization (equal); data curation (lead); formal analysis (equal); investigation (equal); methodology (equal); project administration (equal); resources (equal); validation (equal); visualization (equal); writing – original draft (equal); writing – review and editing (equal).

## CONFLICT OF INTEREST STATEMENT

The authors declare no competing interests.

## Data Availability

The data that support the findings of this study are in the Appendix.

## Appendix

**TABLE A1:** Canarian checklist of ants.

Taxa	Status	El Hierro	La Palma	La Gomera	Tenerife	Gran Canaria	Fuerteventura	Lanzarote
*Aphaenogaster hesperia* Santschi, 1911	Endemic species				X			
*Aphaenogaster iberica* Emery, 1908	Introduced species				X	X		
*Aphaenogaster senilis* Mayr, 1853	Native species					X	X	
*Brachymyrmex cordemoyi* Forel, 1895	Introduced species				X	X		
*Camponotus brullei* (Smith, 1858)	Endemic species				X		X	X
*Camponotus feae* Emery, 1882	Endemic species	X		X	X	X	X	X
*Camponotus guanchus* Santschi, 1908	Endemic species	X	X	X	X			
*Camponotus hesperius* Emery, 1893	Endemic species	X	X	X	X	X	X	
*Cardiocondyla emeryi* Forel, 1881	Introduced species	X	X	X	X	X	X	X
*Cardiocondyla mauritanica* Forel, 1890	Introduced species	X	X	X	X	X	X	X
*Cardiocondyla obscurior* Wheeler, 1929	Introduced species	X		X	X			
*Crematogaster alluaudi* Emery, 1893	Endemic species	X	X	X	X			
*Crematogaster auberti levithorax* Forel, 1902	Native subspecies					X		
*Crematogaster laestrygon canariensis* Barquín Diez, 1981	Endemic subspecies							X
*Hypoponera eduardi* (Forel, 1894)	Native species	X	X	X	X	X		
*Hypoponera nivariana* (Santschi, 1908)	Endemic species		X		X			
*Hypoponera punctatissima* (Roger, 1859)	Introduced species		X		X			X
*Hypoponera ragusai* (Emery, 1894)	Native species	X	X			X		
*Lasius grandis* Forel, 1909	Native species		X	X	X	X		
*Lasius mauretanicus* Seifert, 2020	Native species		X	X	X			
*Lasius neglectus* Van Loon et al., 1990	Introduced species				X			
*Lepisiota capensis* (Mayr, 1862)	Introduced species		X	X		X		
*Lepisiota frauenfeldi* (Mayr, 1855)	Introduced species			X	X		X	X
*Linepithema humile* (Mayr, 1868)	Introduced species	X	X	X	X	X	X	X
*Messor minor hesperius* Santschi, 1927	Endemic subspecies			X	X	X	X	X
*Monomorium hesperium* Emery, 1895	Endemic species		X		X	X	X	X
*Monomorium medinae* Forel, 1892	Endemic species	X			X		X	
*Monomorium pharaonis* (Linnaeus, 1758)	Introduced species	X			X			
*Monomorium salomonis* (Linnaeus, 1758)	Native species			X	X			
*Monomorium subopacum* (Smith, 1858)	Native species	X	X	X	X	X	X	X
*Monomorium wilsoni* Espadaler, 2007	Endemic species	X						
*Myrmoxenus birgitae* (Schulz, 1994)	Endemic species				X			
*Nylanderia jaegerskioeldi* (Mayr, 1904)	Introduced species	X	X	X	X	X	X	X
*Oxyopomyrmex insularis* Santschi, 1908	Endemic species				X			
*Paratrechina longicornis* (Latreille, 1802)	Introduced species	X	X	X	X	X	X	X
*Pheidole bilimeki* Mayr, 1870	Introduced species			X	X			
*Pheidole indica* Mayr, 1879	Introduced species	X	X	X	X	X	X	X
*Pheidole megacephala* (Fabricius, 1793)	Introduced species	X	X	X	X	X	X	X
*Pheidole navigans* Forel, 1901	Introduced species				X			
*Pheidole pallidula* (Nylander, 1849)	Introduced species					X		X
*Plagiolepis alluaudi* Emery, 1894	Introduced species		X		X	X		
*Plagiolepis barbara* Santschi, 1911	Native species	X	X	X	X	X		
*Plagiolepis schmitzii* Forel, 1895	Native species	X	X	X	X	X	X	X
*Solenopsis canariensis* Forel, 1893	Endemic species	X	X	X	X	X	X	X
*Solenopsis geminata* (Fabricius, 1804)	Introduced species					X		
*Strumigenys membranifera* Emery, 1869	Introduced species			X	X	X		
*Tapinoma darioi* Seifert et al., 2017	Introduced species				X			
*Tapinoma erraticum* (Latreille, 1798)	Native species					X		
*Tapinoma melanocephalum* (Fabricius, 1793)	Introduced species	X	X	X	X	X	X	
*Technomyrmex pallipes* (Smith, 1876)	Introduced species					X		
*Temnothorax bimbache* Espadaler, 2007	Endemic species	X				X		
*Temnothorax cabrerae* (Forel, 1893)	Native species			X	X	X		
*Temnothorax canescens* (Santschi, 1908)	Endemic species				X			X
*Temnothorax gracilicornis* (Emery, 1882)	Endemic species	X	X	X	X			
*Temnothorax hesperius* (Santschi, 1909)	Native species			X	X	X		X
*Temnothorax productus* (Santschi, 1918)	Native species				X	X	X	
*Temnothorax risii* (Forel, 1892)	Endemic species	X			X	X		X
*Temnothorax rottenbergii* (Emery, 1870)	Native species	X						X
*Tetramorium bicarinatum* (Nylander, 1846)	Introduced species				X	X		X
*Tetramorium caldarium* (Roger, 1857)	Introduced species	X	X	X	X	X	X	X
*Tetramorium depressum* Forel, 1892	Native species	X	X	X	X	X	X	X

*Note*: The status and the insular distribution of each island are indicated (Gobierno de Canarias, 2024, personal observation).

**TABLE A2:** Known distribution of *Camponotus guanchus* and *Crematogaster alluaudi* based on data of the collection (col) of Pérez‐Delgado and iNaturalist.

No.	Sample code/link	Island	Locality	Lat	Lon	Altitude	Date	Species/color‐morph	Specimens	Source	Syntaxon	Potential vegetation units	Sympatric occurrences
1	2709_LP‐087	LP	Casas Taburiente	28.724129	−17.878776	841	23/6/2001	*Crematogaster alluaudi*	16	col	*Echio plantaginei‐Galactition tomentosae*	Pine forest	
2	147_TF‐095	TF	Malpaís de Rasca	28.017506	−16.694487	40	14/9/2007	*Crematogaster alluaudi*	1	col	*Ceropegio fuscae‐Euphorbietum balsamiferae*	*Euphorbia* scrub and shrublands	
3	917_TF‐042	TF	Zapata, Anaga	28.535499	−16.296191	891	07/01/2013	*Crematogaster alluaudi noualhieri*	1	col	*Myrico fayae‐Ericetum arboreae*	Laurel forest	
4	1032_TF‐014	TF	Montaña Mallorquines, Izaña	28.306596	−16.514383	2245	4/6/2015	*Crematogaster alluaudi*	3	col	*Spartocytisetum supranubii*	High mountain scrub	
5	910_TF‐006	TF	La Mareta	28.224052	−16.612291	2229	20/4/2016	*Crematogaster alluaudi*	1	col	*Spartocytisetum supranubii*	High mountain scrub	
6	111_TF‐059	TF	Los Palacios	28.404260	−16.386068	1031	21/4/2016	*Crematogaster alluaudi*	1	col	*Sideritido solutae‐Pinetum canariensis cistetosum monspeliensis*	Pine forest	
7	107_TF‐062	TF	Recreational area Los Brezos	28.374790	−16.412675	859	28/4/2016	*Crematogaster alluaudi*	4	col	*Sideritido solutae‐Pinetum canariensis cistetosum monspeliensis*	Thermo‐sclerophyllous woodland	
8	911_TF‐004	TF	Cementerio de Tajinastes	28.20886	−16.633019	2078	28/4/2016	*Crematogaster alluaudi*	2	col	*Spartocytisetum supranubii*	High mountain scrub	
9	205_TF‐018	TF	Cuevas Negras	28.254854	−16.700846	2180	4/5/2016	*Crematogaster alluaudi*	1	col	*Spartocytisetum supranubii*	High mountain scrub	
10	204_TF‐016	TF	Montaña Blanca	28.260658	−16.602251	2378	4/5/2016	*Crematogaster alluaudi*	1	col	*Spartocytisetum supranubii*	High mountain scrub	
11	913_TF‐005	TF	Valle de Ucanca	28.21114	−16.618138	2369	4/5/2016	*Crematogaster alluaudi*	1	col	*Erysimo scoparii‐Pterocephaletum lasiospermi*	High mountain scrub	
12	93_TF‐031	TF	Chamorga	28.581653	−16.149212	242	15/5/2016	*Camponotus guanchus* (reddish morph)	1	col	*Artemisio thusculae‐Rumicetum lunariae*	Thermo‐sclerophyllous woodland	X
13	91_TF‐031	TF	Chamorga	28.581653	−16.149212	242	15/5/2016	*Crematogaster alluaudi*	3	col	*Artemisio thusculae‐Rumicetum lunariae*	Thermo‐sclerophyllous woodland	X
14	35_LP‐001	LP	Torreón‐Tamanca	28.556333	−17.878865	469	17/5/2016	*Crematogaster alluaudi*	2	col	*Euphorbio lamarckii‐Retametum rhodorhizoidis*	Thermo‐sclerophyllous woodland	
15	60_LP‐002	LP	Pinos‐Tamanca	28.550826	−17.873127	571	19/5/2016	*Crematogaster alluaudi*	2	col	*Stereocauletum vesuviani*, *Cheilanthion pulchellae*, *Kleinio‐Euphorbietalia* and *Artemisio‐Rumicion* pioneers	Euphorbia scrub and shrublands	
15	TF_Macdiv4	TF	Afur_1	28.55723053	−16.25117113	156	05/05/2017	*Crematogaster alluaudi noualhieri*	15	col	*Artemisio thusculae‐Rumicetum lunariae*	Thermo‐sclerophyllous woodland	X
16	921_TF‐003	TF	Fuente Joco	28.367528	−16.464567	1950	21/5/2016	*Crematogaster alluaudi*	6	col	*Sideritido solutae‐Pinetum canariensis*, *Adenocarpus viscosus* facies	Thermo‐sclerophyllous woodland	
17	MSS_MBT2	TF	Montaña Blanca	28.260423	−16.601483	2352	22/07/2016	*Crematogaster alluaudi*	5	col	*Erysimo scoparii‐Pterocephaletum lasiospermi*	High mountain scrub	
18	327_TF‐071	TF	Punta del Hidalgo_1	28.571767	−16.317327	44	10/8/2016	*Crematogaster alluaudi noualhieri*	4	col	Crop area	*Euphorbia* scrub and shrublands	
19	873_TF‐120	TF	Güímar, Aguerche	28.264102	−16.455739	944	7/9/2016	*Crematogaster alluaudi*	4	col	*Cistetum symphytifolio‐monspeliensis*	Pine forest	
20	699_TF‐129	TF	Malpais de Güímar	28.314978	−16.380135	132	10/9/2016	*Crematogaster alluaudi*	5	col	*Periploco laevigatae‐Euphorbietum canariensis*, *Euphorbia lamarckii* facies	*Euphorbia* scrub and shrublands	
21	850_TF‐267	TF	Arguayo, Santiago del Teide	28.265543	−16.80738	884	13/10/2016	*Crematogaster alluaudi*	30	col	Crop area	Thermo‐sclerophyllous woodland	
22	TF_Macdiv1	TF	Barranco del Tomadero	28.56938254	−16.31565108	57	01/05/2017	*Crematogaster alluaudi noualhieri*	3	col	*Artemisio thusculae‐Rumicetum lunariae*, *Opuntia* spp. facies	Thermo‐sclerophyllous woodland	X
23	TF_Macdiv1	TF	Barranco del Tomadero	28.56938254	−16.31565108	57	01/05/2017	*Camponotus guanchus* (blackish morph)	1	col	*Artemisio thusculae‐Rumicetum lunariae*, *Opuntia* spp. facies	*Euphorbia* scrub and shrublands	X
24	TF_Macdiv2	TF	Pico los cardos	28.56410242	−16.31661791	97	01/05/2017	*Camponotus guanchus* (blackish morph)	4	col	*Artemisio thusculae‐Rumicetum lunariae*, *Opuntia* spp. facies	*Euphorbia* scrub and shrublands	X
25	TF_Macdiv2	TF	Pico los cardos	28.56410242	−16.31661791	97	01/05/2017	*Crematogaster alluaudi noualhieri*	9	col	*Artemisio thusculae‐Rumicetum lunariae*, *Opuntia* spp. facies	Thermo‐sclerophyllous woodland	X
26	TF_Macdiv3	TF	Chinamada_1	28.56336483	−16.29289177	570	04/05/2017	*Camponotus guanchus* (blackish morph)	2	col	*Artemisio thusculae‐Rumicetum lunariae*	Thermo‐sclerophyllous woodland	X
27	TF_Macdiv3	TF	Chinamada_1	28.56336483	−16.29289177	570	04/05/2017	*Crematogaster alluaudi noualhieri*	11	col	*Artemisio thusculae‐Rumicetum lunariae*	Thermo‐sclerophyllous woodland	X
28	TF_Macdiv4	TF	Afur_1	28.55723053	−16.25117113	156	05/05/2017	*Camponotus guanchus* (blackish morph)	1	col	*Artemisio thusculae‐Rumicetum lunariae*	Thermo‐sclerophyllous woodland	X
30	TF_Macdiv5	TF	El Draguillo	28.57631489	−16.18013375	161	06/05/2017	*Camponotus guanchus* (blackish morph)	1	col	*Periploco laevigatae‐Euphorbietum canariensis euphorbietosum balsamiferae*	*Euphorbia* scrub and shrublands	
31	LG_Macdiv1	LG	Barranco de la Concepción	28.08508696	−17.13410776	304	08/05/2017	*Crematogaster alluaudi*	1	col	*Euphorbietum berthelotii*	Thermo‐sclerophyllous woodland	
32	LG_Macdiv3	LG	Lomo de la Montaña	28.08833672	−17.14847801	555	10/05/2017	*Crematogaster alluaudi*	6	col	*Echio plantaginei‐Galactition tomentosae*	Thermo‐sclerophyllous woodland	
33	LG_Macdiv5	LG	El Palmarejo	28.12075402	−17.31818518	683	11/05/2017	*Crematogaster alluaudi*	3	col	Anthropic palm grove	Thermo‐sclerophyllous woodland	
34	2069_LG‐019	LG	La Gollada de Juan de Lugo	28.08876	−17.201467	923	15/5/2017	*Crematogaster alluaudi*	8	col	*Euphorbietum berthelotii‐canariensis*	*Euphorbia* scrub and shrublands	
35	2051_LG‐017	LG	Lomada del Camello	28.085995	−17.136028	314	15/5/2017	*Crematogaster alluaudi*	7	col	*Euphorbietum bertheloti*	Thermo‐sclerophyllous woodland	
36	2104_LG‐027	LG	Presa de Garabato	28.16935	−17.249587	449	7/11/2017	*Crematogaster alluaudi*	7	col	*Rubo‐Salicetum canariensis*	Thermo‐sclerophyllous woodland	
37	2783_H‐023	EH	La Palmita	27.821289	−17.965386	365	28/11/2017	*Crematogaster alluaudi*	1	col	*Rubio fruticosae‐Juniperetum canariensis*	Thermo‐sclerophyllous woodland	
38	1987_H‐052	EH	Cruce Faro Orchilla con el Vertero	27.723458	−18.144665	289	3/2/2018	*Crematogaster alluaudi*	15	col	*Rubio fruticosae‐Euphorbietum balsamiferae*	*Euphorbia* scrub and shrublands	
39	1985_H‐053	EH	Ermita de los Reyes_1	27.729449	−18.120701	686	3/2/2018	*Crematogaster alluaudi*	4	col	Crop area	Thermo‐sclerophyllous woodland	X
40	1989_H‐106	EH	La fuente de Isora	27.749097	−17.941728	757	3/2/2018	*Crematogaster alluaudi*	1	col	*Artemisio thusculae‐Rumicetum lunariae*	Pine forest	
41	2604_LP‐094	LP	Barranco del Agua	28.724824	−17.760765	478	3/7/2018	*Camponotus guanchus* (reddish morph)	1	col	*Myrico fayae‐Ericetum arboreae*	Laurel forest	
42	2391_LP‐069	LP	Tamanca_1	28.551062	−17.872632	584	10/8/2018	*Crematogaster alluaudi*	4	col	*Stereocauletum vesuviani*, *Cheilanthion pulchellae*, *Kleinio‐Euphorbietalia* and *Artemisio‐Rumicion* pioneers	*Euphorbia* scrub and shrublands	X
43	2983_LP‐069	LP	Tamanca_1	28.551062	−17.872632	584	10/8/2018	*Camponotus guanchus* (reddish morph)	1	col	*Stereocauletum vesuviani*, *Cheilanthion pulchellae*, *Kleinio‐Euphorbietalia* and *Artemisio‐Rumicion* pioneers	*Euphorbia* scrub and shrublands	X
44	2468_H‐053	EH	Ermita de los Reyes_1	27.729449	−18.120701	686	13/8/2018	*Camponotus guanchus* (reddish morph)	1	col	Crop area	Thermo‐sclerophyllous woodland	X
45	2446_H‐078	EH	Mirador de Isora	27.738273	−17.951393	826	14/8/2018	*Crematogaster alluaudi*	5	col	Crop area	Thermo‐sclerophyllous woodland	
46	2453_H‐065	EH	Mirador de la Peña	27.807376	−17.980601	645	14/8/2018	*Crematogaster alluaudi*	17	col	Crop area	Pine forest	
47	2485_H‐088	EH	Charco Azul	27.761346	−18.038443	65	15/8/2018	*Crematogaster alluaudi*	4	col	*Euphorbio lamarckii‐Schizogynetum sericeae*	*Euphorbia* scrub and shrublands	
48	2501_H‐089	EH	La Frontera	27.752154	−18.014603	269	15/8/2018	*Camponotus guanchus* (reddish morph)	1	col	Urban area	Thermo‐sclerophyllous woodland	
49	3135_TF‐357	TF	Direccion Palmar	28.357657	−16.843022	345	24/4/2019	*Crematogaster alluaudi noualhieri*	2	col	*Periploco laevigatae‐Euphorbietum canariensis*	*Euphorbia* scrub and shrublands	
50	3046_TF‐343	TF	Teno Bajo	28.347279	−16.911819	127	24/4/2019	*Crematogaster alluaudi*	1	col	*Junipero canariensis‐Oleetum cerasiformis*, *Euphorbia lamarckii* facies	Thermo‐sclerophyllous woodland	
51	2834_TF‐309	TF	Barranco Cochinos	28.343534	−16.817421	461	5/8/2019	*Crematogaster alluaudi noualhieri*	7	col	*Visneo mocanerae‐Arbutetum canariensis*	Laurel forest	
52	2986_TF‐330	TF	Hoyo Hondo	28.052596	−16.551666	60	7/2/2021	*Crematogaster alluaudi*	1	col	Crop area	*Euphorbia* scrub and shrublands	
53	2998_LP‐097	LP	El Poris, Monte de Luna	28.531003	−17.796481	168	2/3/2021	*Crematogaster alluaudi*	27	col	*Artemisio thusculae‐Rumicetum lunariae*, *Euphorbia lamarckii* facies	*Euphorbia* scrub and shrublands	X
54	2999_LP‐097	LP	El Poris, Monte de Luna	28.531003	−17.796481	168	2/3/2021	*Camponotus guanchus* (reddish morph)	2	col	Euphorbio lamarckii‐Retametum rhodorhizoidis	Thermo‐sclerophyllous woodland	X
55	2868_LP‐096	LP	Tamanca_2	28.558228	−17.873706	627	2/3/2021	*Crematogaster alluaudi*	4	col	*Artemisio thusculae‐Rumicetum lunariae*, *Euphorbia lamarckii* facies	*Euphorbia* scrub and shrublands	X
56	2864_LP‐096	LP	Tamanca_2	28.558228	−17.873706	627	2/3/2021	*Camponotus guanchus* (reddish morph)	4	col	*Euphorbio lamarckii‐Retametum rhodorhizoidis*	Thermo‐sclerophyllous woodland	X
57	3010_LP‐098	LP	Salemera	28.57455	−17.768131	148	3/3/2021	*Camponotus guanchus* (reddish morph)	1	col	*Rhamno crenulatae‐Hypericetum canariensis*, *Heberdenia excelsa* facies	Thermo‐sclerophyllous woodland	
58	2919_H‐110	EH	El Golfo	27.749081	−18.027184	358	23/3/2021	*Crematogaster alluaudi*	11	col	*Rhamno crenulatae‐Hypericetum canariensis*	Thermo‐sclerophyllous woodland	X
59	2918_H‐110	EH	El Golfo	27.749081	−18.027184	358	23/3/2021	*Camponotus guanchus* (reddish morph)	3	col	*Rhamno crenulatae‐Hypericetum canariensis*	Thermo‐sclerophyllous woodland	X
60	2921_H‐111	EH	Ermita de los Reyes_2	27.725525	−18.117804	682	24/3/2021	*Crematogaster alluaudi*	23	col	*Euphorbio lamarckii‐Schizogynetum sericeae*	*Euphorbia* scrub and shrublands	
61	2997_H‐112	EH	La Restiga	27.65996	−17.975586	183	24/3/2021	*Crematogaster alluaudi*	2	col	*Euphorbio lamarckii‐Schizogynetum sericeae*	*Euphorbia* scrub and shrublands	
62	3004_H‐113	EH	La Pared Caida, Valverde	27.790717	−17.916303	428	24/3/2021	*Camponotus guanchus* (reddish morph)	3	col	*Rubio fruticosae‐Juniperetum canariensis*, *Euphorbia lamarckii* facies	Thermo‐sclerophyllous woodland	
63	2915_H‐109	EH	Barranco de la Charca	27.750295	−18.141589	308	24/3/2021	*Crematogaster alluaudi*	4	col	*Artemisio thusculae‐Rumicetum lunariae*	Thermo‐sclerophyllous woodland	
64	2872_TF‐318	TF	Mirador de Humboldt	28.408009	−16.507249	322	25/3/2021	*Camponotus guanchus* (blackish morph)	1	col	*Artemisio thusculae‐Rumicetum lunariae*	Thermo‐sclerophyllous woodland	
65	2910_LG‐089	LG	Tamargada	28.195501	−17.247864	113	6/4/2021	*Crematogaster alluaudi*	10	col	*Euphorbietum berthelotii‐canariensis*, *Euphorbia balsamiferae* facies	*Euphorbia* scrub and shrublands	
66	2907_LG‐092	LG	Hermigua	28.153997	−17.198209	216	7/4/2021	*Camponotus guanchus* (reddish morph)	1	col	Crop area	Thermo‐sclerophyllous woodland	
67	2906_TF‐322	TF	Barranco las Huertas, San Andres	28.52513	−16.191642	109	13/4/2021	*Camponotus guanchus* (reddish morph)	2	col	Urban area	Thermo‐sclerophyllous woodland	
68	2927_TF‐323	TF	Ucanca	28.22916	−16.641113	2132	24/4/2021	*Crematogaster alluaudi*	1	col	*Spartocytisetum supranubii*	High mountain scrub	
69	3033_TF‐342	TF	Tegueste	28.523957	−16.333327	407	24/6/2021	*Crematogaster alluaudi noualhieri*	1	col	Crop area	Thermo‐sclerophyllous woodland	
70	2958_TF‐327	TF	Punta del Hidalgo_2	28.572548	−16.318717	53	17/7/2021	*Crematogaster alluaudi noualhieri*	15	col	*Artemisio thusculae‐Rumicetum lunariae*, *Opuntia* spp. facies	*Euphorbia* scrub and shrublands	X
71	2954_TF‐327	TF	Punta del Hidalgo_2	28.572548	−16.318717	53	17/7/2021	*Camponotus guanchus* (blackish morph)	9	col	*Artemisio thusculae‐Rumicetum lunariae*, *Opuntia* spp. facies	*Euphorbia* scrub and shrublands	X
72	2963_TF‐329	TF	Pedro Alvarez	28.530315	−16.308788	628	1/8/2021	*Crematogaster alluaudi noualhieri*	19	col	*Artemisio thusculae‐Rumicetum lunariae*, *Opuntia* spp. facies	Laurel forest	
73	3025_TF‐341	TF	Mirador de Amogoje	28.558669	−16.205923	415	10/10/2021	*Crematogaster alluaudi noualhieri*	1	col	*Myrico fayae‐Ericetum arboreae*	Laurel forest	X
74	3024_TF‐341	TF	Mirador de Amogoje	28.558669	−16.205923	415	10/10/2021	*Camponotus guanchus* (blackish morph)	1	col	*Myrico fayae‐Ericetum arboreae*	Laurel forest	X
75	3052_TF‐345	TF	Bajamar	28.554536	−16.339611	69	19/11/2021	*Camponotus guanchus* (reddish morph)	1	col	Urban area	Thermo‐sclerophyllous woodland	
76	3055_TF‐346	TF	Afur_2	28.56277	−16.25323	87	21/11/2021	*Crematogaster alluaudi noualhieri*	3	col	*Artemisio thusculae‐Rumicetum lunariae*	*Euphorbia* scrub and shrublands	
77	3070_TF‐347	TF	Chinamada_2	28.562829	−16.295155	546	12/12/2021	*Camponotus guanchus* (blackish morph)	1	col	*Artemisio thusculae‐Rumicetum lunariae*	Thermo‐sclerophyllous woodland	X
78	3071_TF‐347	TF	Chinamada_2	28.562829	−16.295155	546	12/12/2021	*Crematogaster alluaudi noualhieri*	3	col	*Artemisio thusculae‐Rumicetum lunariae*	Thermo‐sclerophyllous woodland	X
79	3140_TF‐361	TF	Pista Madre del Agua2	28.167273	−16.62925	1621	01/05/2022	*Crematogaster alluaudi*	12	col	*Sideritido solutae‐Pinetum canariensis*	Pine forest	
80	3289_TF‐393	TF	Barranco de Ruiz	28.38603	−16.627542	267	13/05/2023	*Crematogaster alluaudi noualhieri*	2	col	*Artemisio thusculae‐Rumicetum lunariae*	Thermo‐sclerophyllous woodland	
81	3219_TF‐379	TF	Las Raices_1	28.419543	−16.377018	968	14/05/2023	*Crematogaster alluaudi noualhieri*	15	col	*Sideritido solutae‐Pinetum canariensis ericetosum arboreae*	Pine forest	
82	3302_H‐116	EH	Montaña de los Guirres	27.751168	−18.141962	297	25/05/2023	*Crematogaster alluaudi*	3	col	*Euphorbio lamarckii‐Schizogynetum sericeae*	*Euphorbia* scrub and shrublands	
83	3236_LP‐110	LP	Los Charcos, Fuencaliente	28.535808	−17.872798	510	30/05/2023	*Camponotus guanchus* (reddish morph)	7	col	Euphorbio lamarckii‐Retametum rhodorhizoidis	Thermo‐sclerophyllous woodland	
84	3248_TF‐387	TF	Las Raices_2	28.418049	−16.377356	955	09/07/2023	*Crematogaster alluaudi*	6	col	*Sideritido solutae‐Pinetum canariensis ericetosum arboreae*	Pine forest	
85	3272_H‐115	EH	Mirador de Lomo Negro	27.753845	−18.143991	259	23/07/2023	*Crematogaster alluaudi*	5	col	*Euphorbio lamarckii‐Schizogynetum sericeae*	*Euphorbia* scrub and shrublands	X
86	3273_H‐115	EH	Mirador de Lomo Negro	27.753845	−18.143991	259	23/07/2023	*Camponotus guanchus* (reddish morph)	2	col	*Euphorbio lamarckii‐Schizogynetum sericeae*	*Euphorbia* scrub and shrublands	X
87	3275_H‐116	EH	La Dehesa	27.751168	−18.141962	297	24/07/2023	*Crematogaster alluaudi*	1	col	Urban area	Thermo‐sclerophyllous woodland	
88	3309_H‐117	EH	Fuga de Gorreta	27.781752	−17.995003	326	26/07/2023	*Camponotus guanchus* (reddish morph)	13	col	*Rubio fruticosae‐Juniperetum canariensis*, *Euphorbia lamarckii* facies	Thermo‐sclerophyllous woodland	X
89	3308_H‐117	EH	Fuga de Gorreta	27.781752	−17.995003	326	26/07/2023	*Crematogaster alluaudi*	25	col	*Rubio fruticosae‐Juniperetum canariensis*, *Euphorbia lamarckii* facies	Thermo‐sclerophyllous woodland	X
90	3271_TF‐388	TF	Barranco Hondo	28.415759	−16.383277	1043	30/07/2023	*Crematogaster alluaudi*	2	col	*Sideritido solutae‐Pinetum canariensis ericetosum arboreae*	Pine forest	
91	https://www.iN.org/observations/141906119	TF	Benijo	28.573114	−16.182973	245		*Crematogaster alluaudi noualhieri*	1	iN	*Artemisio thusculae‐Rumicetum lunariae*	*Euphorbia* scrub and shrublands	
92	https://www.iN.org/observations/161863483	TF	Las Poyatas	28.574385	−16.319634	19		*Crematogaster alluaudi noualhieri*	2	iN	Crop area	*Euphorbia* scrub and shrublands	
93	https://www.iN.org/observations/182977597	TF	Punta del Hidalgo_4	28.571577	−16.316902	38		*Crematogaster alluaudi noualhieri*	1	iN	Crop area	*Euphorbia* scrub and shrublands	
94	https://www.iN.org/observations/170088691	TF	Punta del Hidalgo_3	28.571727	−16.317098	39		*Crematogaster alluaudi noualhieri*	1	iN	Crop area	*Euphorbia* scrub and shrublands	
95	https://www.iN.org/observations/163872073	TF	Mirador Punta del Fraile	28.36487	−16.881578	145		*Crematogaster alluaudi*	1	iN	*Ceropegio dichotomae‐Euphorbietum aphyllae*	*Euphorbia* scrub and shrublands	
96	https://www.iN.org/observations/109739953	TF	Barranco de Ruiz	28.389115	−16.627147	180		*Crematogaster alluaudi noualhieri*		iN	*Artemisio thusculae‐Rumicetum lunariae*	Thermo‐sclerophyllous woodland	
97	https://www.iN.org/observations/124917082	TF	Barranco la Cruz_1	28.432421	−16.491536	198		*Crematogaster alluaudi noualhieri*		iN	Artemisio thusculae‐Rumicion lunariae	Thermo‐sclerophyllous woodland	
98	https://www.iN.org/observations/137411119	TF	Barranco la Cruz_2	28.431635	−16.490202	206		*Crematogaster alluaudi noualhieri*		iN	Artemisio thusculae‐Rumicion lunariae	Thermo‐sclerophyllous woodland	
99	https://www.iN.org/observations/123572204	TF	Barranco la Cruz_3	28.431647	−16.491062	211		*Crematogaster alluaudi noualhieri*		iN	Artemisio thusculae‐Rumicion lunariae	Thermo‐sclerophyllous woodland	
100	https://www.iN.org/observations/178600314	TF	Barranco la Cruz_4	28.430324	−16.489235	226		*Crematogaster alluaudi noualhieri*		iN	Artemisio thusculae‐Rumicion lunariae	Thermo‐sclerophyllous woodland	
101	https://www.iN.org/observations/134747490	TF	Mirador Punta del Fraile	28.365862	−16.884312	234		*Crematogaster alluaudi noualhieri*		iN	*Ceropegio dichotomae‐Euphorbietum aphyllae*	*Euphorbia* scrub and shrublands	
102	https://www.iN.org/observations/193067382	TF	Rambla de Castro	28.396193	−16.594354	95		*Camponotus guanchus* (blackish morph)	1	iN	Crop area	Thermo‐sclerophyllous woodland	
103	https://www.iN.org/observations/176134264	TF	Buenavista	28.358668	−16.838727	313		*Crematogaster alluaudi noualhieri*		iN	*Rhamno crenulatae‐Hypericetum canariensis*, *Heberdenia excelsa* facies	Thermo‐sclerophyllous woodland	
104	https://www.iN.org/observations/139245523	TF	Bajamar	28.543298	−16.337742	320		*Crematogaster alluaudi noualhieri*		iN	*Periploco laevigatae‐Euphorbietum canariensis*	*Euphorbia* scrub and shrublands	X
105	https://www.iN.org/observations/190085243	TF	Tejina	28.543298	−16.337742	320		*Crematogaster alluaudi noualhieri*		iN	*Periploco laevigatae‐Euphorbietum canariensis*	*Euphorbia* scrub and shrublands	
106	https://www.iN.org/observations/135661501	TF	Barranco de Herqués	28.245646	−16.429161	333		*Crematogaster alluaudi*		iN	*Euphorbietum atropurpureae*	Thermo‐sclerophyllous woodland	
107	https://www.iN.org/observations/122621646	TF	La degollada, Tegueste	28.530844	−16.338726	478		*Crematogaster alluaudi noualhieri*		iN	*Artemisio thusculae‐Rumicetum lunariae*, *Opuntia* spp. facies	Laurel forest	
108	https://www.iN.org/observations/122621506	TF	La Ensillada, Tegueste	28.529395	−16.32771	535		*Crematogaster alluaudi noualhieri*		iN	*Pinus radiata*	Pine forest	
109	https://www.iN.org/observations/41533012	TF	Tafada	28.578493	−16.149031	554		*Crematogaster alluaudi noualhieri*	2	iN	*Myrico fayae‐Ericetum arboreae*	Laurel forest	
110	https://www.iN.org/observations/122621508	TF	Fuente de la Mocanera, Tegueste	28.530048	−16.327821	563		*Crematogaster alluaudi noualhieri*		iN	*Artemisio thusculae‐Rumicetum lunariae*, *Opuntia* spp. facies	Laurel forest	
111	https://www.iN.org/observations/139852917	TF	Barranco del Rey	28.100754	−16.69136	599		*Crematogaster alluaudi*		iN	*Periploco laevigatae‐Euphorbietum canariensis*	*Euphorbia* scrub and shrublands	
112	https://www.iN.org/observations/189386456	TF	San Roque	28.488914	−16.30858	641		*Crematogaster alluaudi*		iN	*Artemisio thusculae‐Rumicetum lunariae*, *Opuntia* spp. facies	Laurel forest	
113	https://www.iN.org/observations/150669661	TF	Jardina	28.524948	−16.281079	772		*Crematogaster alluaudi noualhieri*		iN	*Artemisio thusculae‐Rumicetum lunariae*	Thermo‐sclerophyllous woodland	
114	https://www.iN.org/observations/139890907	TF	Roque del Conde	28.106098	−16.697026	843		*Crematogaster alluaudi*		iN	*Periploco laevigatae‐Euphorbietum canariensis*	*Euphorbia* scrub and shrublands	
115	https://www.iN.org/observations/103920702	TF	Las Barreras	28.423269	−16.364643	851		*Crematogaster alluaudi noualhieri*		iN	Urban area	Thermo‐sclerophyllous woodland	
116	https://www.iN.org/observations/181466940	TF	Cruz del Carmen	28.530333	−16.280456	954		*Crematogaster alluaudi noualhieri*		iN	*Ilici canariensis‐Ericetum platycodonis*	Laurel forest	
117	https://www.iN.org/observations/160054057	TF	Barranco las Pasadillas	28.42202	−16.380751	1024		*Crematogaster alluaudi*		iN	*Sideritido solutae‐Pinetum canariensis ericetosum arboreae*	Pine forest	
118	https://www.iN.org/observations/186729976	LG	Reventón Oscuro	28.123198	−17.209869	1034		*Crematogaster alluaudi*		iN	*Ilici canariensis‐Ericetum platycodonis*	Laurel forest	
119	https://www.iN.org/observations/115207788	TF	Barranco de Tenazo	28.25188	−16.477556	1066		*Crematogaster alluaudi*		iN	*Sideritido solutae‐Pinetum canariensis*, *Chamaecytisus proliferus* facies	Pine forest	
120	https://www.iN.org/observations/152840370	TF	Las Raíces_3	28.425217	−16.382613	1072		*Crematogaster alluaudi noualhieri*		iN	*Sideritido solutae‐Pinetum canariensis ericetosum arboreae*	Pine forest	
121	https://www.iN.org/observations/134038207	TF	Mirador de Vilaflor	28.174831	−16.640512	1690		*Crematogaster alluaudi*		iN	*Sideritido solutae‐Pinetum canariensis*	Pine forest	
122	https://www.iN.org/observations/116844306	TF	Fuente Joco	28.368052	−16.464817	1950		*Crematogaster alluaudi*		iN	*Pinus canariensis*	Pine forest	
123	https://www.iN.org/observations/116966909	TF	Fuente Joco	28.368314	−16.46485	1953		*Crematogaster alluaudi*		iN	*Pinus canariensis*	Pine forest	
124	https://www.iN.org/observations/105443013	TF	Cerca del Mirador de las Narices	28.255207	−16.699864	2186		*Crematogaster alluaudi*		iN	*Spartocytisetum supranubii*	High mountain scrub	
125	https://www.iN.org/observations/2659674	TF	Cañadas del Teide	28.25035	−16.620941	2269		*Crematogaster alluaudi*		iN	*Spartocytisetum supranubii*	High mountain scrub	
126	https://www.iN.org/observations/139369227	TF	Bajamar	28.543298	−16.337742	320		*Camponotus guanchus* (blackish morph)		iN	*Periploco laevigatae‐Euphorbietum canariensis*	*Euphorbia* scrub and shrublands	X
127	https://www.iN.org/observations/116106415	TF	El Tejal_1	28.571699	−16.317012	38		*Camponotus guanchus* (blackish morph)		iN	Crop area	*Euphorbia* scrub and shrublands	
128	https://www.iN.org/observations/182977596	TF	El Tejal_2	28.571597	−16.316886	41		*Camponotus guanchus* (blackish morph)		iN	Crop area	*Euphorbia* scrub and shrublands	
129	https://www.iN.org/observations/167560954	TF	El Tejal_3	28.571834	−16.31727	35		*Camponotus guanchus* (blackish morph)		iN	Crop area	*Euphorbia* scrub and shrublands	

*Note*: Localities with data on geographic coordinates, altitude, date of collection, habitat information, (syntaxon and potential vegetation Units) (del Arco Aguilar & Delgado, 2018), and cases of syntopic occurrence are given. Island: Tenerife (TF), La Palma (LP), La Gomera (LG), and El Hierro (EH).

## References

[ece370113-bib-0001] Baroni Urbani, C. (1969). Trail sharing between *Camponotus* and *Cremastogaster*: Some comments and ideas. Proceedings of the 6th Congress of the International Union for the Study of Social Insects, VI 11–17.

[ece370113-bib-0002] Barquín Diez, J. (1981). Las hormigas de Canarias. Taxonomia, ecologia y distribucion de los Formicidae. In Secretariado de publicaciones de la Universidad de La Laguna, Tenerife (p. 584). Universidad de La Laguna.

[ece370113-bib-0003] Bates, H. W. (1861). Contributions to an insect fauna of the Amazon valley. Lepidoptera: Heliconidae. Transactions of the Linnean Society, 23, 495–566.

[ece370113-bib-0004] Benes, E. S. (1969). Behavioral evidence for color discrimination by the whiptail lizard, *Cnemidophorus tigris* . Copeia, 4, 707–722.

[ece370113-bib-0005] Blaimer, B. B. (2012). Acrobat ants go global – Origin, evolution and systematics of the genus *Crematogaster* (Hymenoptera: Formicidae). Molecular Phylogenetics and Evolution, 65, 421–436. 22796480 10.1016/j.ympev.2012.06.028

[ece370113-bib-0006] Borovsky, V. , Borovsky, R. , & Borovsky, M. (2022). Beitrag zur Biologie von Camponotus lateralis (Hymenoptera, Formicidae), einer Ameisenart, die in Österreich bisher (noch) nicht gefunden wurde (pp. 11–30). Carinthia II 212./132.

[ece370113-bib-0007] Bračko, G. (2023). Atlas of the ants of Slovenia (p. 251). Biotechnical Faculty.

[ece370113-bib-0008] Buren, W. F. (1959). A review of the species of Crematogaster, sensu stricto, in North America (Hymenoptera: Formicidae) part I. Journal of the New York Entomological Society, 66, 119–134.

[ece370113-bib-0009] Cagniant, H. (1996). Les Camponotus du Maroc (Hymenoptera: Formicidae): Clé et catalogue des espèces. Annales de la Societe Entomologique de France, 32, 87–100.

[ece370113-bib-0010] Cagniant, H. , & Espadaler, X. (1993). *Camponotus guanchus* Santschi, 1908, stat. nov. et étude des populations de *Camponotus sicheli* Mayr, 1866 (Hymenoptera: Formicidae). Journal of African Zoology, 107, 419–438.

[ece370113-bib-0011] Carpintero, S. , Reyes‐López, J. , & Arias de Reyna, L. (2005). Impact of Argentine ants (*Linepithema humile*) on an arboreal ant community in Doñana National Park, Spain. Biodiversity and Conservation, 14, 151–163.

[ece370113-bib-0012] Crans, G. G. , & Shuster, J. J. (2008). How conservative is Fisher's exact test? A quantitative evaluation of the two‐sample comparative binomial trial. Statistics in Medicine, 27, 3598–3611. 18338319 10.1002/sim.3221

[ece370113-bib-0013] Daloze, D. , Braekman, J.‐C. , Vanhecke, P. , Boevé, J.‐L. , & Pasteels, J. M. (1987). Long chain electrophilic contact poisons from the Dufour's gland of the ant *Crematogaster scutellaris* (Hymenoptera, Myrmicinae). Canadian Journal of Chemistry, 65, 432–436.

[ece370113-bib-0014] Daloze, D. , Kaisin, M. , Detrain, C. , & Pasteels, J. M. (1991). Chemical defense in the three European species of *Crematogaster* ants. Experientia, 47, 1082–1089.

[ece370113-bib-0015] Darwin, C. R. (1859). On the origin of species by means of natural selection, or the preservation of favoured races in the struggle for life (1st ed., p. 502). John Murray. PMC5184128 30164232

[ece370113-bib-0016] Darwin, C. R. (1871). The descent of man, and selection in relation to sex (p. 450). John Murray.

[ece370113-bib-0017] De Los Santos, A. , & De Nicolás, J. P. (2008). Environmental niche of the smut lizard population on a sandy coastal ecosystem of southeastern Tenerife (Canary Islands). Marine Ecology, 29, 2–11.

[ece370113-bib-0018] del Arco Aguilar, M. J. , & Delgado, O. R. (2018). Vegetation of the Canary Islands (p. 429). Springer.

[ece370113-bib-0019] Emery, C. (1886). Mimetismo e costumi parassitari del *Camponotus lateralis* Ol. Bullettino Della Società Entomologica Italiana, 18, 412–413.

[ece370113-bib-0020] Emery, C. (1893). Voyage de M Ch. Alluaud aux îles Canaries. Formicides. Annales de la Societe Entomologique de France, 62, 81–88.

[ece370113-bib-0021] Emery, C. (1925). I *Camponotus* (*Myrmentoma*) paleartici del gruppo *Lateralis* . Rendiconti Delle Sessioni Della Reale Accademia Delle Scienze dell'Istituto di Bologna, 29, 62–72.

[ece370113-bib-0022] Farina, B. , & Aguilar, N. (2002). Nota sobre la población de *Gallotia galloti* (Oudart, 1839) (Lacertidae) de Roque de Fasnia (Tenerife, Islas Canarias). Revista de la Academia Canaria de Ciencias, 14, 305–309.

[ece370113-bib-0023] Fisher, B. L. , & Peeters, C. (2019). Fourmis de Madagascar: Un guide pour les 62 genres = ants of Madagascar: A guide to the 62 genera (p. 260). Association Vahatra.

[ece370113-bib-0024] Fisher, R. A. (1922). On the interpretation of χ2 from contingency tables, and the calculation of P. Journal of the Royal Statistical Society, 85, 87–94.

[ece370113-bib-0025] Fisher, R. A. (1925). Statistical methods for research workers (1st ed., p. 239). Oliver & Boyd.

[ece370113-bib-0026] Fisher, R. A. (1930). The Genetical theory of natural selection (p. 272). Oxford University Press.

[ece370113-bib-0027] Forel, A.‐H. (1874). Les fourmis de la Suisse. Systématique, notices anatomiques et physiologiques, architecture, distribution géographique, nouvelles expériences et observations de moeurs (p. 452). Allgemeine Schweizerische Gesellschaft für die Gesamten Naturwissenschaften.

[ece370113-bib-0028] Forel, A.‐H. (1891). Les Formicides. In: Histoire Physique, Naturelle et Politique de Madagascar. In Histoire naturelle des Hyménoptères (pp. 1–231). Hachette et Cie.

[ece370113-bib-0029] Franks, D. W. , & Noble, J. (2004). Batesian mimics influence mimicry ring evolution. Proceedings of the Royal Society of London, Series B: Biological Sciences, 271, 191–196. 10.1098/rspb.2003.2582 PMC1691580 15058397

[ece370113-bib-0030] Gallego Ropero, M. C. , & Feitosa, R. M. (2014). Evidences of Batesian mimicry and parabiosis in ants of the Brazilian Savannah. Sociobiology, 61, 281–285.

[ece370113-bib-0031] Gillespie, R. G. , & Roderick, G. K. (2002). Arthropods on islands: Colonization, speciation, and conservation. Annual Review of Entomology, 47, 595–632. 10.1146/annurev.ento.47.091201.145244 11729086

[ece370113-bib-0032] Gobierno de Canarias . (2024). Banco de datos de biodiversidad de canarias. Gobierno de Canarias.

[ece370113-bib-0033] Gobin, B. , Peeters, C. , Billen, J. , & Morgan, E. D. (1998). Interspecific trail following and commensalism between the ponerine ant *Gnamptogenys menadensis* and the formicine ant *Polyrhachis rufipes* . Journal of Insect Behaviour, 11, 361–368.

[ece370113-bib-0034] Goetsch, W. (1942). Beiträge zur Biologie spanischer Ameisen. Eos Revista Española de Entomología, 18, 175–241.

[ece370113-bib-0035] Goetsch, W. (1951). Ameisen‐und Termiten‐Studien in Ischia Capri und Neapel. Zoologische Jahrbücher Abteilung für Systematik Ökologie Und Geographie der Tiere, 80, 64–98.

[ece370113-bib-0036] Graham, N. R. , Gruner, D. S. , Lim, J. Y. , & Gillespie, R. G. (2017). Island ecology and evolution: Challenges in the Anthropocene. Environmental Conservation, 44, 323–335.

[ece370113-bib-0037] Hess, C. (1917). Der Farbensinn der Vögel und die Lehre von den Schmuckfarben. Pflüger's Archiv für Die Gesamte Physiologie Des Menschen Und der Tiere, 166, 381–426.

[ece370113-bib-0038] Hölldobler, B. , & Wilson, E. O. (1990). The ants (p. 732). The Belknap Press of Harvard University Press.

[ece370113-bib-0039] Ito, F. , Hashim, R. , Huei, Y. S. , Kaufmann, E. , Akino, T. , & Billen, J. (2004). Spectacular Batesian mimicry in ants. Naturwissenschaften, 91, 481–484. 15729761 10.1007/s00114-004-0559-z

[ece370113-bib-0040] Kaudewitz, F. (1955). Zum Gastverhältnis zwischen *Crematogaster scutellaris* Ol. mit *Camponotus lateralis bicolor* Ol. Biologisches Centralblatt, 74, 69–87.

[ece370113-bib-0041] Kikuchi, D. W. , & Pfennig, D. W. (2010). High‐model abundance may permit the gradual evolution of Batesian mimicry: An experimental test. Proceedings of the Royal Society B: Biological Sciences, 277, 1041–1048. 10.1098/rspb.2009.2000 PMC2842773 19955153

[ece370113-bib-0042] Kikuchi, D. W. , & Pfennig, D. W. (2013). Imperfect mimicry and the limits of natural selection. The Quarterly Review of Biology, 88, 297–315. 24552099 10.1086/673758

[ece370113-bib-0043] Komárek, S. (2002). Mimicry, aposematism, and related phenomena (p. 167). Lincom München.

[ece370113-bib-0044] Kunte, K. , Kizhakke, A. G. , & Nawge, V. (2021). Evolution of mimicry rings as a window into community dynamics. Annual Review of Ecology, Evolution, and Systematics, 52, 315–341.

[ece370113-bib-0045] Lamon, B. , & Topoff, H. (1981). Avoiding predation by army ants: Defensive behaviours of three ant species of the genus *Camponotus* . Animal Behaviour, 29, 1070–1081.

[ece370113-bib-0046] Lashley, K. S. (1916). The color vision of birds I. The spectrum of the domestic fowl. Journal of Animal Behavior, 6, 1–26.

[ece370113-bib-0047] Mallet, J. , & Gilbert, L. E. (1995). Why are there so many mimicry rings? Correlations between habitat, behaviour and mimicry in *Heliconius* butterflies. Biological Journal of the Linnean Society, 55, 159–180.

[ece370113-bib-0048] Marlier, J. F. , Quinet, Y. , & de Biseau, J. C. (2004). Defensive behaviour and biological activities of the abdominal secretion in the ant *Crematogaster scutellaris* (Hymenoptera: Myrmicinae). Behavioural Processes, 67, 427–440. 15518992 10.1016/j.beproc.2004.07.003

[ece370113-bib-0049] McIver, J. D. (1987). On the myrmecomorph *Coquillettia insignis* Uhler (Hemiptera: Miridae): Arthropod predators as operators in an ant‐mimetic system. Zoological Journal of the Linnean Society, 90, 133–144.

[ece370113-bib-0050] Menzel, F. , Orivel, J. , Kaltenpoth, M. , & Schmitt, T. (2014). What makes you a potential partner? Insights from convergently evolved ant‐ant symbioses. Chemoecology, 24, 105–119.

[ece370113-bib-0051] Menzel, F. , Woywod, M. , Blüthgen, N. , & Schmitt, T. (2010). Behavioural and chemical mechanisms behind a Mediterranean ant‐ant association. Ecological Entomology, 35, 711–720.

[ece370113-bib-0052] Merrill, D. N. , & Elgar, M. A. (2000). Red legs and golden gasters: Batesian mimicry in Australian ants. Naturwissenschaften, 87, 212–215. 10883435 10.1007/s001140050705

[ece370113-bib-0053] Montgomery, G. G. (1985). Movements, foraging and food habits of the four extant species of neotropical vermilinguas (Mammalia; Myrmecophagidae). In G. G. Montgomery (Ed.), The evolution and ecology of armadillos, sloths, and vermilinguas (pp. 365–377). Smithsonian Institution Press.

[ece370113-bib-0054] Müller, F. (1879). *Ituna* and *Thyridia*; a remarkable case of mimicry in butterflies. Transactions of the Entomological Society of London, Xx–Xxix.

[ece370113-bib-0055] Novosolov, M. , & Meiri, S. (2013). The effect of Island type on lizard reproductive traits. Journal of Biogeography, 40, 2385–2395.

[ece370113-bib-0056] Oliveira, P. S. , & Sazima, I. (1984). The adaptive bases of ant‐mimicry in a neotropical aphantochilid spider (Araneae: Aphantochilidae). Biological Journal of the Linnean Society, 22, 145–155.

[ece370113-bib-0057] Padilla, D. P. , Nogales, M. , & Pérez, A. J. (2005). Seasonal diet of an insular endemic population of southern Grey shrike *Lanius meridionalis koenigi* on Tenerife, Canary Islands. Ornis Fennica, 82, 155–165.

[ece370113-bib-0058] Pekár, S. (2020). Ant‐mimicking spider actively selects its mimetic model (Araneae: Gnaphosidae; Hymenoptera: Formicidae). Myrmecological News, 30, 131–137.

[ece370113-bib-0059] Pekár, S. , & Křál, J. (2002). Mimicry complex in two central European zodariid spiders (Araneae: Zodariidae): How *Zodarion* deceives ants. Biological Journal of the Linnean Society, 75, 517–532.

[ece370113-bib-0060] Pekár, S. , Petráková, L. , Bulbert, M. W. , Whiting, M. J. , & Herberstein, M. E. (2017). The golden mimicry complex uses a wide spectrum of defence to deter a community of predators. eLife, 6, e22089. 28170317 10.7554/eLife.22089 PMC5295815

[ece370113-bib-0061] Pfennig, D. W. , & Mullen, S. P. (2010). Mimics without models: Causes and consequences of allopatry in Batesian mimicry complexes. Proceedings of the Royal Society B: Biological Sciences, 277, 2577–2585. 10.1098/rspb.2010.0586 PMC2982051 20484238

[ece370113-bib-0062] Pie, M. R. , & Del‐Claro, K. (2002). Male‐male agonistic behavior and ant‐mimicry in a Neotropical Richardiid (Diptera: Richardiidae). Studies on Neotropical Fauna and Environment, 37, 19–22.

[ece370113-bib-0063] Poulton, E. B. (1890). The colours of animals their meaning and use (p. 360). Kegan Paul, Trench & Trübner.

[ece370113-bib-0064] Powell, S. , Del‐Claro, K. , Feitosa, R. M. , & Brandão, C. R. F. (2014). Mimicry and eavesdropping enable a new form of social parasitism in ants. The American Naturalist, 184, 500–509. 10.1086/677927 25226185

[ece370113-bib-0065] QGIS Development Team . (2019). QGIS geographic information system. Open Source Geospatial Foundation Project. http://qgis.osgeo.org

[ece370113-bib-0066] Quicke, D. L. J. (2017). Mimicry, crypsis, masquerade and other adaptive resemblances (p. 576). John Wiley & Sons.

[ece370113-bib-0067] Rasekh, A. , Michaud, J. P. , Kharazi‐Pakdel, A. , & Allahyari, H. (2010). Ant mimicry by an aphid parasitoid *Lysiphlebus fabarum* . Journal of Insect Science, 10, 1–14. 20879920 10.1673/031.010.12601 PMC3016887

[ece370113-bib-0068] Rasoamanana, N. , Csősz, S. , & Fisher, B. L. (2017). Taxonomic revision of imitating carpenter ants, *Camponotus* subgenus *Myrmopytia* (Hymenoptera, Formicidae) of Madagascar, using morphometry and qualitative traits. ZooKeys, 681, 119–152. 10.3897/zookeys.681.13187 PMC5523882 28769722

[ece370113-bib-0069] Reiskind, J. (1977). Ant‐mimicry in Panamanian clubionid and salticid spiders (Araneae: Clubionidae, Salticidae). Biotropica, 9, 1–8.

[ece370113-bib-0070] Ribes, J. , Pagola‐Carte, S. , & Heiss, E. (2008). Two new Phylinae (Hemiptera: Heteroptera: Miridae) from the Canary Islands. Acta Entomologica Musei Nationalis Pragae, 48, 423–431.

[ece370113-bib-0071] Roca‐Cusachs, M. , & Goula, M. (2016). New genus and species of ant‐like true bug (Hemiptera: Miridae) from the Canary Islands. Zootaxa, 4173, 66–74. 27701204 10.11646/zootaxa.4173.1.6

[ece370113-bib-0072] Roca‐Cusachs, M. , Suárez, D. , Osorio, V. , García‐Becerra, R. , García‐Pérez, J. , Hernández‐Teixidor, D. , Pérez‐Delgado, A. J. , Pérez‐Valcárcel, J. , París, M. , Oromí, P. , & Goula, M. (2020). Updated check‐list and geographic database of new chorological data of true bugs (Insecta, Hemiptera, Heteroptera) from the Canary Islands. Arquivos Entomolóxicos, 22, 169–218.

[ece370113-bib-0073] Rodríguez, A. , Nogales, M. , Rumeu, B. , & Rodríguez, B. (2008). Temporal and spatial variation in the diet of the endemic lizard *Gallotia galloti* in an insular Mediterranean scrubland. Journal of Herpetology, 42, 213–222.

[ece370113-bib-0074] Rodríguez, A. , Rodríguez, B. , Rumeu, B. , & Nogales, M. (2007). Seasonal diet of the Grey heron *Ardea cinerea* on an oceanic Island (Tenerife, Canary Islands): Indirect interaction with wild seed plants. Acta Ornithologica, 42, 77–87.

[ece370113-bib-0075] Ruxton, G. D. , Sherratt, T. N. , & Speed, M. P. (2004). Avoiding attack: The evolutionary ecology of crypsis, warning signals and mimicry (p. 260). Oxford University Press.

[ece370113-bib-0076] Santschi, F. (1908). Nouvelles fourmis de l'Afrique du nord. Annales de la Societe Entomologique de France, 77, 517–534.

[ece370113-bib-0077] Santschi, F. (1919). Fourmis d'Espagne et des., Canaries. Boletin de la Real Sociedad Espanola de Historia Natural, 19, 241–248.

[ece370113-bib-0078] Santschi, F. (1937). Contribution à l'étude des *Crematogaster* paléarctiques. Mémoires de la Société Vaudoise Des Sciences Naturelles, 5, 295–317.

[ece370113-bib-0079] Schifani, E. , Giannetti, D. , Csősz, S. , Castellucci, F. , Luchetti, A. , Spotti, F. A. , Castracani, C. , Mori, A. , & Grasso, D. A. (2022). Is mimicry as a diversification driver in ants? Biogeography, ecology, ethology, genetics and morphology define a second west‐Palaearctic *Colobopsis* species (Hymenoptera: Formicidae). Zoological Journal of the Linnean Society, 20, 1–27.

[ece370113-bib-0080] Seifert, B. (2009). Lebensraumansprüche und Erreichbarkeit für Spechte relevanter Ameisen Biomassen. Schriftenreihe Aus Dem Nationalpark Harz, 3, 20–27.

[ece370113-bib-0081] Seifert, B. (2018). The ants of central and North Europe (p. 408). Lutra Verlags‐ und Vertriebsgesellschaft.

[ece370113-bib-0082] Seifert, B. (2019). A taxonomic revision of the members of the *Camponotus lateralis* species group (Hymenoptera: Formicidae) from Europe, Asia minor and Caucasia. Soil Organisms, 91, 7–32.

[ece370113-bib-0083] Speed, M. P. (1999). Batesian, quasi‐Batesian or Müllerian mimicry? Theory and data in mimicry research. Evolutionary Ecology, 13, 755–776.

[ece370113-bib-0084] Symula, R. , Schulte, R. , & Summers, K. (2001). Molecular phylogenetic evidence for a mimetic radiation in Peruvian poison frogs supports a Müllerian mimicry hypothesis. Proceedings of the Royal Society of London. Series B: Biological Sciences, 268, 2415–2421. 10.1098/rspb.2001.1812 PMC1088895 11747559

[ece370113-bib-0085] Taniguchi, K. , Maruyama, M. , Ichikawa, T. , & Ito, F. (2005). A case of Batesian mimicry between a myrmecophilous staphylinid beetle, *Pella comes*, and its host ant, *Lasius* (*Dendrolasius*) *spathepus*: An experiment using the Japanese treefrog, *Hyla japonica* as a Real Predator. Insectes Sociaux, 52, 320–322.

[ece370113-bib-0086] The Heliconius Genome Consortium . (2012). Butterfly genome reveals promiscuous exchange of mimicry adaptations among species. Nature, 487, 94–98. 22722851 10.1038/nature11041 PMC3398145

[ece370113-bib-0087] Valido, A. , & Nogales, M. (2003). Digestive ecology of two omnivorous Canarian lizard species (*Gallotia*, Lacertidae). Amphibia‐Reptilia, 24, 331–344.

[ece370113-bib-0088] Valido, A. , Nogales, M. , & Medina, F. M. (2003). Fleshy fruits in the diet of Canarian lizards *Gallotia galloti* (Lacertidae) in a xeric habitat of the Island of Tenerife. Journal of Herpetology, 37, 741–747.

[ece370113-bib-0089] Wagner, H. (1932). Über den Farbensinn der Eidechsen. Zeitschrift für Vergleichende Physiologie, 18, 378–392.

[ece370113-bib-0090] Wagner, H. C. (2013). Gedanken zur Evolution der Rotfärbung bei Ameisen und erste Hinweise auf eine Ameisen‐Ameisen‐Mimikry aus Europa (Hymenoptera: Formicidae). Entomologica Austriaca, 20, 225.

[ece370113-bib-0091] Wagner, H. C. (2014). Die Ameisen Kärntens. Verbreitung, Biologie, Ökologie und Gefährdung (p. 462). Naturwissenschaftlicher Verein für Kärnten.

[ece370113-bib-0092] Wagner, H. C. (2019). Wiener Ameisenbeobachtungen (Hymenoptera: Formicidae). Beiträge zur Entomofaunistik, 20, 143–159.

[ece370113-bib-0093] Wallace, A. R. (1866). On the phenomena of variation and geographical distribution, as illustrated by the Papilionidae of the Malayan region. Transactions of the Linnean Society of London, 35, 1–72.

[ece370113-bib-0094] Ward, P. S. (1984). A revision of the ant genus *Rhytidoponera* (Hymenoptera: Formicidae) in New Caledonia. Australien Journal of Zoology, 32, 131–175.

[ece370113-bib-0095] Ward, P. S. (2009). The ant genus *Tetraponera* in the afrotropical region: The *T. grandidieri* group (Hymenoptera: Formicidae). Journal of Hymenoptera Research, 18, 285–304.

[ece370113-bib-0096] Wellenius, O. H. (1955). Entomologische Ergebnisse der finnländischen Kanaren‐Expedition 1947–1951. No. 10. Formicidae Insularum Canariensium. Systematik, Ökologie und Verbreitung der Kanarischen Formiciden. Commentationes Biologicae, 15, 1–20.

[ece370113-bib-0097] Whittaker, R. J. , & Fernandez‐Palacios, J. M. (2007). Island biogeography: Ecology, evolution, and conservation (p. 401). Oxford University Press.

[ece370113-bib-0098] Wickler, W. (1968). Mimicry in plants and animals (p. 255). Weidenfeld & Nicolson.

[ece370113-bib-0099] Wilson, J. S. , Jahner, J. P. , Forister, M. L. , Sheehan, E. S. , Williams, K. A. , & Pitts, J. P. (2015). North American velvet ants form one of the world's largest known Müllerian mimicry complexes. Current Biology, 25, R704–R706. 26294178 10.1016/j.cub.2015.06.053

[ece370113-bib-0100] Yerkes, R. M. , & Eisenberg, A. M. (1915). Preliminaries to a study of color vision in the ring‐dove *Turtur risorius* . Journal of Animal Behavior, 5, 25–43.

[ece370113-bib-0101] Zurek, D. B. , Cronin, T. W. , Taylor, L. A. , Byrne, K. , Sullivan, M. L. G. , & Morehouse, N. I. (2015). Spectral filtering enables trichromatic vision in colorful jumping spiders. Current Biology, 25, R403–R404. 25989075 10.1016/j.cub.2015.03.033

